# Modifiable lifestyle factors and cancer risk in Latin America: A systematic review with meta-analysis

**DOI:** 10.1016/j.isci.2026.117381

**Published:** 2026-09-05

**Authors:** Guillermo Barahona-Fuentes, Leandro F.M. Rezende, Jacqueline Wahrhaftig, Paloma Ferrero-Hernández, Eduardo Nilson, Gerson Ferrari

**Affiliations:** 1 Universidad de Santiago de Chile (USACH), Escuela de Ciencias de la Actividad Física, el Deporte y la Salud, Santiago, Chile; 2 Chronic Disease Epidemiology Research Center, Department of Preventive Medicine, Universidade Federal de São Paulo, Sao Paulo, Brazil; 3 Facultad de Ciencias de la Salud, Universidad Autónoma de Chile, Santiago, Chile; 4 Vicerrectoría de Investigación e Innovación, Universidad Arturo Prat, Iquique, Chile

**Keywords:** neoplasms, risk factors, Latin America, public health, epidemiology

## Abstract

Cancer risk in Latin America is shaped by lifestyle exposures whose regional patterns are usually represented through extrapolated global estimates. We systematically reviewed observational studies on tobacco smoking, alcohol consumption, adiposity, physical inactivity, and dietary patterns and cancer risk in Latin American adults, searching four databases through September 2025 and appraising risk of bias with QUIPS. Forty-seven studies were included, almost all case-control. Pooling was restricted to site-specific sets sharing one exposure contrast, and only two qualified: a higher dietary inflammatory index with prostate cancer (OR 1.38, 95% CI 1.11–1.73) and obesity with endometrial cancer (OR 3.81, 1.08–13.45). In a direction-of-effect synthesis, healthy dietary exposures were protective in 9 of 9 estimates, whereas smoking and unhealthy diets were predominantly associated with increased risk. These associations are directional rather than causal, and they indicate where prospective regional cohorts are most needed.

## Highlights

• First Latin American synthesis of lifestyle factors and cancer risk (47 studies)
• Only two site-specific pools held: DII-prostate and obesity-endometrial cancer
• Healthy diet was protective in all 9 contributing estimates, across six sites
• Evidence is mostly case-control, limiting causal certainty; cohorts are needed

## Teaser

Medicine; Oncology; Public health

## Introduction

Cancer is one of the leading causes of morbidity and mortality worldwide.^1^ Recent global estimates indicate that approximately 37.8% of new cancer cases are attributable to modifiable risk factors, with smoking, infections, and alcohol consumption acting as primary drivers.^2^ In Latin America, approximately 1.4 million new cancer cases and 670,000 deaths are recorded each year; against this backdrop, five-year survival remains lower than in high-income countries.^3^^,^^4^ At the regional level, cancer has been associated with a double burden: malignancies linked to infections, such as cervical cancer related to human papillomavirus and gastric cancer associated with *Helicobacter pylori*, as well as tumors linked to lifestyle factors, including breast, colorectal, and lung cancers.^5^ This dual epidemiologic burden underscores the need to analyze lifestyle risk factors in Latin America.

In this context, in several Latin American countries the prevalence of tobacco smoking exceeds 15%,^6^ and it frequently co-occurs with occupational exposures and nutritional deficiencies that can substantially potentiate carcinogen activation in the lung and other sites.^7^ At the same time, obesity affects more than 25% of the adult population and has grown in parallel with a dietary transition that replaces fresh and minimally processed foods with ultra-processed foods,^8^^,^^9^ thereby fostering chronic inflammatory processes and hormonal alterations related to cancer.^10^ Alcohol consumption varies by beverage type and drinking patterns, which can modulate its carcinogenic effects across populations.^11^ For example, global adult per-capita alcohol consumption rose from 5.9 to 6.5 L of pure alcohol between 1990 and 2017 and is projected to reach 7.6 L by 2030.^12^ In addition, more than 40% of Latin American adults do not meet minimum physical-activity recommendations, which has been associated with several cancer sites.^13^

Despite the high prevalence and co-occurrence of these risk factors across the region, the available evidence remains fragmented. While global burden studies provide high-level estimates, they often rely on extrapolations that may not fully capture the specific exposure patterns or the heterogeneity of study designs found in Latin America. Consequently, the lack of a rigorous, systematic synthesis of local data limits the evidence base required to design effective, context-specific public policies.^4^^,^^14^

Herein, we aimed to perform a systematic review and meta-analysis to identify and synthesize the direction and—where poolable data were available—the strength of association between modifiable lifestyle factors (tobacco smoking, alcohol consumption, adiposity, physical inactivity, and dietary patterns) and cancer at various sites among adults in Latin America.

## Results

**Figure 1:**
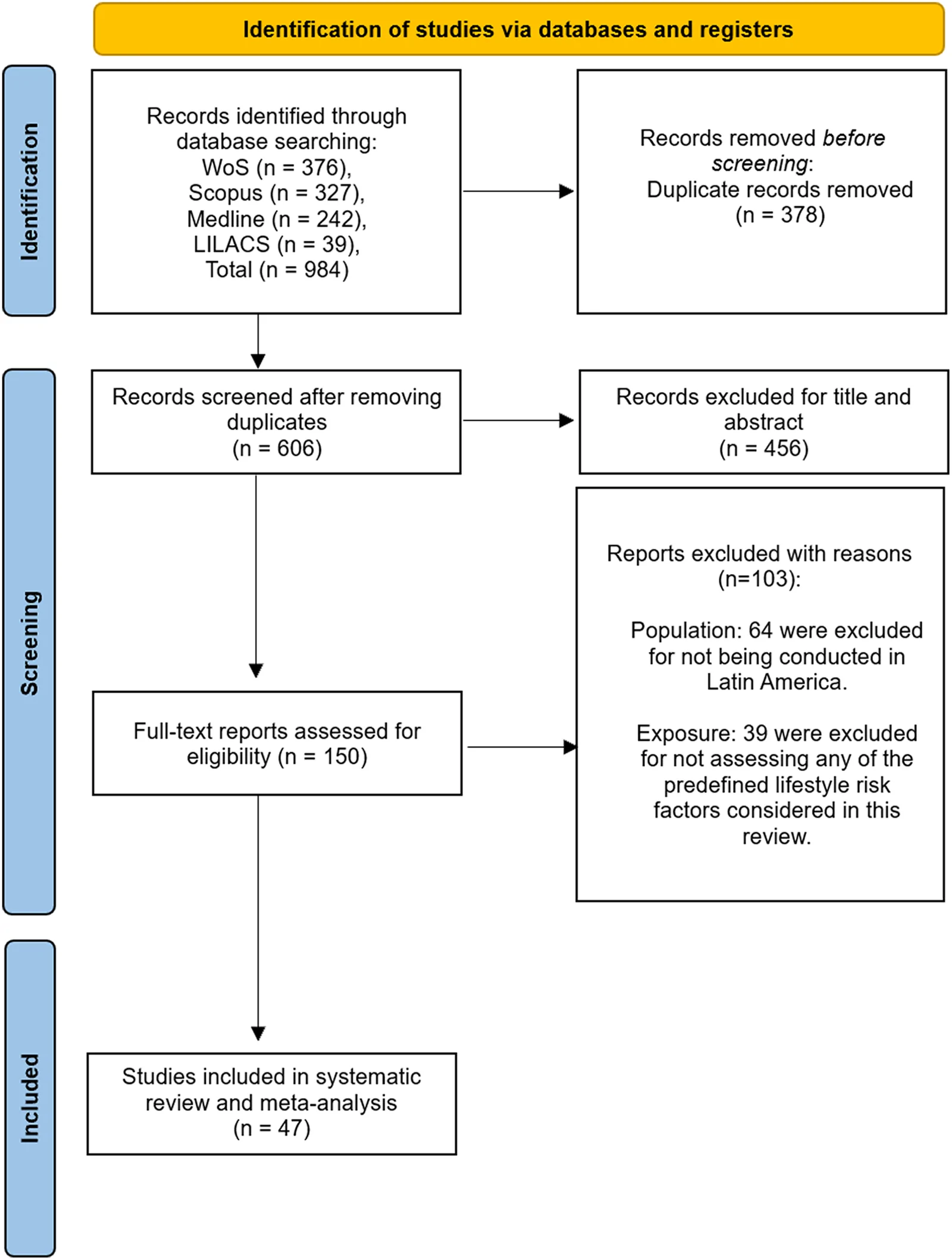
PRISMA 2020 flow diagram of study identification, screening, eligibility assessment, and inclusion (984 records identified; 47 studies included)

The search strategy yielded a total of 984 records, of which 378 were duplicates and were subsequently removed. The remaining 606 records were screened by title and abstract, resulting in 150 studies selected for full-text review. After applying the inclusion criteria, 103 studies were excluded. A total of 47 studies were ultimately included in the systematic review and meta-analysis (Figure 1).

A total of 47 studies published between 1987 and 2023 were included (references 15–61). Studies were conducted in Mexico (*n* = 24), Brazil (*n* = 10), Argentina (*n* = 4), Uruguay (*n* = 1), Chile (*n* = 1), and Peru (*n* = 1)—plus multicenter collaborations that spanned Argentina-Brazil-Uruguay-Paraguay (*n* = 2), Brazil-Argentina-Cuba (*n* = 2), Costa Rica-Colombia-Mexico-Panama (*n* = 1), and Bolivia-Mexico (*n* = 1). Most estimates were adjusted for potential confounders, albeit with heterogeneous covariate sets; while age and sex were frequently controlled, adjustment for other key confounders (e.g., socioeconomic status, BMI, smoking history) varied substantially by study. The most frequently assessed cancer sites were breast (*n* = 17), gastric (*n* = 7), prostate (*n* = 6), and colorectal (*n* = 5), followed by oral cavity/upper aerodigestive tract (*n* = 4) and endometrial (*n* = 3); lung and esophagus were each assessed in one study, as were ovarian, bladder, cervical, brain, and gallbladder cancers (one study reported three sites). At the estimate level, the direction-of-effect synthesis comprised 46 effect estimates across six behavioral domains—unhealthy diet (16), healthy diet (9), cigarette smoking (7), adiposity (6), alcohol (5), and physical activity (3) (Table 3)—with several studies contributing estimates to more than one domain or site. The detailed characteristics of each study, including specific exposure definitions and adjustment variables, are fully described in Table 1.

**Table 1:** Characteristics of studies included in the systematic review

Author, year	Country	Design (recruitment)	Cases (*n*)	Controls (*n*)	Exposure(s) evaluated	Cancer site	Audited effect estimate (OR, 95% CI)	Contrast/note	Inclusion status	Risk of bias (QUIPS overall)
Amadou^15^	Mexico	Population-based (CAMA multicentre)	1,000	1,074	Body-size/silhouette trajectory (adiposity)	Breast	0.604 (0.46–0.80)	Body-silhouette trajectory; inverse association (premenopausal)	Narrative synthesis	Moderate
Amadou^16^	Mexico	Population-based (CAMA multicentre)	1,000	1,074	Dietary carbohydrate/glycemic index	Breast	1.30 (1.00–1.70)	Highest vs. lowest category	Narrative synthesis	Moderate
Ángeles-Llerenas^17^	Mexico	Population-based (CAMA multicentre)	1,000	1,074	Moderate physical activity	Breast	0.61 (0.40–0.94)	Effect modified by menopausal status (non-collapsible)	Narrative synthesis	Moderate
Beasley^18^	Mexico	Population-based (CAMA multicentre)	1,000	1,074	Alcohol consumption	Breast	1.25 (0.99–1.58)	Ever vs. never drinker	Narrative synthesis	Moderate
Castellsagué^19^	Argentina, Brazil, Paraguay, Uruguay	Population-based (IARC multicentre)	830	1,779	Tobacco smoking; alcohol drinking	Esophageal	Smoking 3.24 (2.40–4.37); Alcohol 3.70 (2.70–4.90)	Standardized from dose-extreme; sex strata collapsed; IARC sample overlap	Narrative synthesis	Low
Coquet^20^	Argentina	Hospital-based	318	526	Traditional dietary pattern	Breast	1.30 (1.00–1.70)	Highest vs. lowest tertile; spurious smoking OR removed	Narrative synthesis	Moderate
Denova-Gutiérrez^21^	Mexico	Population-based	248	478	Healthy & pro-inflammatory dietary patterns	Gastric	Healthy 0.43 (0.24–0.77); Pro-inflammatory 4.80 (2.56–8.98)	Highest vs. lowest tertile	Narrative synthesis	Moderate
Facin^22^	Brazil	Hospital-based	82	165	Physical activity	Colorectal	0.83 (0.31–2.28)	Active vs. inactive	Narrative synthesis	Moderate
Flores-García^23^	Mexico	Population-based	1,045	1,030	Western/Prudent dietary patterns	Breast	Prudent 0.54 (0.44–0.65); Western 21.45 (13.87–33.16)	Menopause-stratified (non-collapsible); premenopausal shown (postmenopausal Prudent 0.55, Western 11.95); Western implausibly large; old 1.62/0.76 absent from source	Narrative synthesis	Moderate
Franco-Marina^24^	Mexico	Population-based (multicentre)	385	898	Active and passive smoking	Lung	4.00 (2.90–5.50)	Ever vs. never, both sexes; standardized from dose-extreme	Narrative synthesis	Moderate
Galván-Portillo^25^	Mexico	Population-based	395	797	Dietary flavonoids (isolated nutrient)	Prostate	0.39 (0.27–0.56)	Highest vs. lowest quartile	Narrative synthesis	Moderate
Giacomazzi^26^	Brazil	Hospital-based	1,007	1,007	Tobacco smoking (ever)	Breast; Colorectal; Prostate	Breast 4.2 (2.4–7.5); CRC 3.1 (1.5–6.3); Prostate 10.5 (4.2–26.3)	Per-site analytic *N* varies; prostate estimate implausibly large	Narrative synthesis	High
Hernández-Pérez^27^	Mexico	Population-based	394	793	Metabolic syndrome	Prostate	1.94 (1.37–2.75)	Present vs. absent	Narrative synthesis	Moderate
Hernández-Ramírez^28^	Mexico	Population-based	257	478	Polyphenols (cinnamic acids), nitrate, nitrite	Gastric	Cinnamic acids 0.61 (0.38–0.97)	T3 vs. T1 (Model 3); shares Mexico-City gastric sample with Denova-Gutiérrez — different exposure, overlap documented	Narrative synthesis (sample overlap flagged)	Moderate
Herrero^29^	Colombia, Costa Rica, Mexico, Panama	Population-based (IARC multicentre)	667	1,430	Cigarette smoking (current)	Cervical	1.00 (0.70–1.20)	Current vs. never; null after adjustment	Narrative synthesis	Moderate
Iscovich^30^	Argentina	Population-based	117	234	Tobacco smoking; occupation	Bladder	4.32 (1.90–10.30)	Ever vs. never; 117 hospital +117 neighborhood controls	Narrative synthesis	Moderate
Jiménez-Mendoza^31^	Mexico	Population-based	402	805	Cigarette smoking (life-course)	Prostate	Overall null; Gleason ≥8 2.30 (1.21–4.38)	Overall prostate cancer not associated; only poorly differentiated (Gleason ≥8) subgroup significant	Narrative synthesis	Moderate
Lajous^32^	Mexico	Population-based	226	699	Folate, vitamin B6/B12 (isolated nutrient)	Breast	0.64 (0.45–0.90)	Highest vs. lowest quartile (folate); analytic subset	Narrative synthesis	Moderate
López-Carrillo^33^	Mexico	Population-based	220	752	Chili pepper consumption	Gastric	5.49 (2.72–11.06)	Any vs. none; corrected from dose-extreme 17.11	Narrative synthesis	Moderate
López-Carrillo^34^	Mexico	Population-based	220	752	Alcohol (wine)	Gastric	2.93 (1.27–6.75)	Highest vs. none; same sample as 1994	Narrative synthesis	Moderate
López-Carrillo^35^	Mexico	Hospital-based	234	468	Capsaicin consumption (with *H*. *pylori*)	Gastric	1.71 (0.76–3.88)	High vs. low	Narrative synthesis	Low
Luz^36^	Brazil	Population-based	49	58	Dietary factors (isolated food)	Brain	1.30 (0.61–2.78)	Highest vs. lowest intake	Narrative synthesis	High
Marchioni^37^	Brazil	Hospital-based	366	469	Processed/cured meat (isolated food)	Oral cavity	1.49 (0.99–2.27)	Highest vs. lowest tertile; *trans*-fat not measured	Narrative synthesis	Moderate
Navarro-Ibarra^38^	Mexico	Hospital-based	81	81	Waist circumference (continuous); weight gain	Breast	0.93 (0.90–0.97) per cm	Per-cm waist (continuous); not comparable to categorical adiposity; inverse direction likely reverse causation	Narrative synthesis (excluded from categorical pool)	High
Okada^39^	Chile	Nested case-control (screening cohort)	195	Unverified	Alcohol; lifestyle	Colorectal	1.16 (0.87–1.55)	Adjusted; corrected from crude age-subgroup; controls count not confirmed	Narrative synthesis	Moderate
Ortiz-Mendoza^40^	Mexico	Hospital-based	22	44	Obesity (BMI ≥ 30)	Endometrial	8.10 (2.46–26.60)	Pilot study (*N* = 66); inflates pooled estimate	Meta-analysis (obesity × endometrial)	High
Pineda^41^	Mexico	Hospital-based	60	Unverified	Caloric dietary pattern; cooking methods	Breast	4.99 (1.39–17.92)	Exploratory; controls count not confirmed in PDF	Narrative synthesis	High
Queiroz^42^	Brazil	Hospital-based	100	178	Ultra-processed foods; excess weight	Breast	UPF 2.35 (1.08–5.12); Excess weight (BMI ≥ 25) 2.70 (1.28–5.70)	Adjusted (not 2.55); “obesity BMI ≥ 30” contrast does not exist	Narrative synthesis	High
Ramírez-Díaz^43^	Mexico	Hospital-based	98	196	Diet/lifestyle (isolated food)	Colorectal	2.79 (1.32–5.89)	Highest vs. lowest category	Narrative synthesis	Moderate
Rodrigues^44^	Brazil	Hospital-based	80	80	Vitamin A/diet (isolated nutrient)	Breast	0.41 (0.20–0.84)	Highest vs. lowest intake; 1:1 matched	Narrative synthesis	Moderate
Ronco^45^	Uruguay	Hospital-based	572	889	Mate and tea intake	Breast	0.38 (0.25–0.58)	Highest vs. lowest intake	Narrative synthesis	Moderate
Salazar-Martínez^46^	Mexico	Population-based	85	668	Physical activity; obesity (BMI ≥ 30)	Endometrial	PA 0.47 (0.26–0.86); Obesity 2.20 (1.20–4.20)	Obesity corrected (3.60 was diabetes, not obesity)	Meta-analysis (obesity × endometrial); PA narrative	Moderate
Salazar-Martínez^47^	Mexico	Population-based	84	629	Vitamin D; dietary fat (isolated nutrient)	Ovarian	Vitamin D 0.43 (0.23–0.80); Total fat 0.60 (0.33–1.06)	“Vitamin E 0.44” was Vitamin D; total fat direction corrected (was 1.91)	Narrative synthesis	Moderate
Salazar-Martínez^48^	Mexico	Population-based	85	629	Dietary fat (animal fat)	Endometrial	1.19 (0.55–2.58)	Corrected from 4.00 (no association); impossible CI fixed	Narrative synthesis	Moderate
Sanabria-Rojas^49^	Peru	Hospital-based	142	143	Vegetarian dietary pattern	Colorectal	0.05 (0.01–0.59)	Vegetarian vs. non-vegetarian; very wide CI	Narrative synthesis	High
Sánchez-Zamorano^50^	Mexico	Population-based (CAMA)	1,000	1,074	Folate intake × testosterone	Breast	Joint 9.18 (2.56–32.88)	Synergistic interaction (low folate + high testosterone); joint effect, not a marginal OR — not poolable	Narrative synthesis	Moderate
Sassano^51^	Multinational (StoP consortium)	Pooled analysis (consortium)	5,362	11,497	Dietary vitamin C (pooled)	Gastric	0.64 (0.58–0.72)	StoP Pooling Project pooled estimate; may overlap included studies; shown for context	Excluded (pooled consortium, not primary)	Low
Shivappa^52^	Argentina	Hospital-based	153	309	Dietary inflammatory index (DII)	Prostate	1.50 (1.24–1.80)	Tertile 3 vs. 1; raw DII	Meta-analysis (DII × prostate)	Moderate
Silva^53^	Brazil	Hospital-based	492	377	Energy-adjusted DII (E-DII)	Gastric	2.60 (1.16–5.70)	Q4 vs. Q1, endoscopy controls; corrected (DII 1.85 did not exist)	Narrative synthesis	Moderate
Strom^54^	Bolivia, Chile, Mexico	Hospital-based (international)	84	126	Obesity (BMI ≥28); lifestyle	Gall bladder	2.60 (0.50–18.60)	Vs. non-stone controls; non-stone control count not confirmed	Narrative synthesis	Moderate
Szymańska^55^	Argentina, Brazil, Cuba, Paraguay, Uruguay	Population-based (IARC multicentre)	252	1,707	Alcohol drinking (ever)	Oral cavity/UADT	4.62 (3.39–6.28)	Ever vs. never; IARC sample overlap	Narrative synthesis	Low
Toledo^56^	Brazil	Population-based	210	251	Prudent dietary pattern	Oral/pharyngeal	0.44 (0.25–0.75)	Highest vs. lowest tertile; IARC sample overlap	Narrative synthesis	Moderate
Toporcov^57^	Brazil	Hospital-based	296	296	Animal-origin foods (isolated food)	Oral cavity	3.04 (1.51–6.15)	Highest vs. lowest intake	Narrative synthesis	Moderate
Torres-Sánchez^58^	Mexico	Hospital-based	198	198	Phytoestrogen food sources (isolated food)	Breast	0.27 (0.16–0.47)	Highest vs. lowest intake; 1:1 matched	Narrative synthesis	Moderate
Torres-Sánchez^59^	Mexico	Hospital-based	141	141	Dietary flavonols/flavones	Breast	0.21 (0.07–0.60)	Postmenopausal subgroup, secondary analysis	Narrative synthesis	Moderate
Tumas^60^	Argentina	Hospital-based	100	294	Traditional dietary pattern	Breast	Traditional 3.13 (2.58–3.78)	T3 vs. T1; shares Coquet 2016 Córdoba sample — non-independent (excluded from pooling only, retained for narrative)	Narrative synthesis (non-independent sample)	Moderate
Vázquez-Salas^61^	Mexico	Population-based	394	794	Energy-adjusted DII (E-DII)	Prostate	1.18 (0.85–1.63)	Tertile 3 vs. 1; energy-adjusted	Meta-analysis (DII × prostate)	Moderate

Lifestyle factors and cancer risk in Latin America. Each cell verified against the primary publication and the audited dataset; audited effect estimates supersede pre-audit values.

Abbreviations: BMI, body mass index; CI, confidence interval; CRC, colorectal cancer; DII, dietary inflammatory index; E-DII, energy-adjusted DII; FFQ, food-frequency questionnaire; IARC, International Agency for Research on Cancer; OR, odds ratio; PA, physical activity; QUIPS, Quality In Prognosis Studies; UADT, upper aerodigestive tract; UPF, ultra-processed foods. CAMA = Mexican multicentre population-based breast-cancer study (Mexico City, Monterrey, Veracruz). Sample sizes are study-level case/control counts; analytic *N* may differ by exposure. “Excluded” rows are listed for completeness and do not contribute estimates.

Methodological quality and risk of bias: the overall risk of bias was low in 40%, moderate in 53%, and high in 6% of studies. At the domain level, outcome measurement was consistently low risk (100%), and statistical analysis/reporting was low in 92% (the remaining 8% were moderate). Study participation was a frequent concern (moderate in 62%, low in 38%), as was exposure measurement (moderate in 66%, high in 2%, low in 32%). Confounding was adequately addressed in 66% of studies (28% moderate; 6% high). Attrition bias was low in 51% and moderate in 49%. A risk-of-bias traffic-light plot (Figure S1) and the detailed per-study QUIPS domain ratings (Table S1) are provided, and a sensitivity analysis restricted to low-risk-of-bias studies preserved the direction of effect for every domain with a clear directional signal; adiposity, already equivocal in the full synthesis (binomial *p* = 0.22), was the only domain whose estimates split evenly under this restriction (Table S5).

### Quantitative and narrative synthesis by lifestyle risk factor

**Figure 2:**
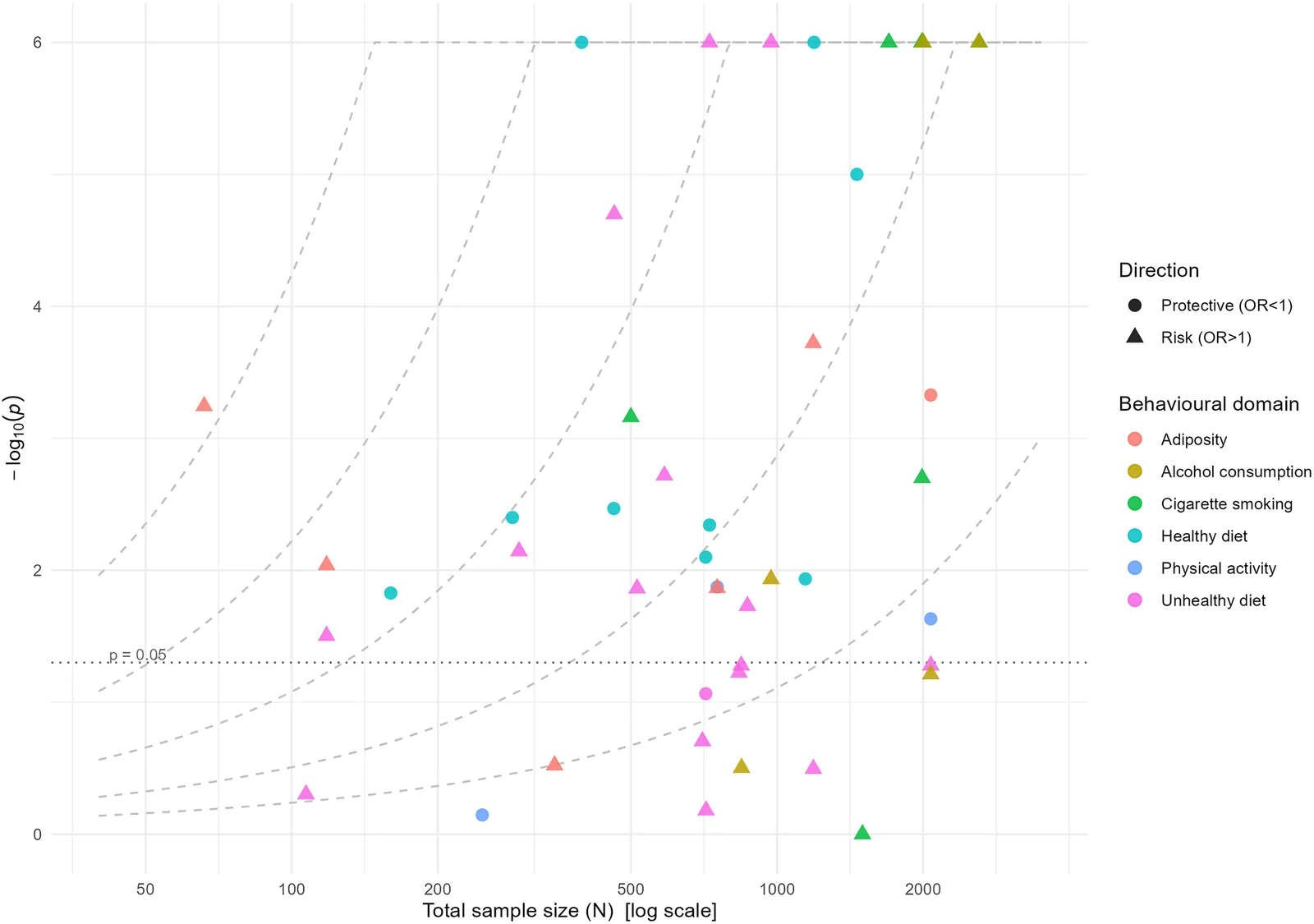
Albatross plot of the whole body of evidence, relating study *p* values to sample size on contours of effect size; healthy dietary exposures cluster on the protective side *p* values are two-sided.

Detailed statistical metrics for all meta-analyses, including tau-squared (τ^2^) and prediction intervals (PIs), are reported in the corresponding tables and text. While a global overview of the regional burden is available in Table S2 and Figure S2 for reference, the following analyses prioritize site-specific associations. The two site-specific pooled estimates are reported in Table 2. The direction-of-effect synthesis across behavioral domains is presented in Table 3, the GRADE certainty of evidence in Table 4, and the albatross plot of the whole evidence base in Figure 2.

**Table 2:** Site-specific pooled estimates (confirmatory)

Pool	k	Studies	OR fixed (95% CI)	OR random (95% CI)	I2	tau2
DII/E-DII x prostate cancer	2	Shivappa^52^; Vázquez-Salas^61^	1.41 (1.20–1.66)	1.38 (1.11–1.73)	36%	0.010
Obesity (BMI>=30) x endometrial cancer	2	Ortiz-Mendoza^40^; Salazar-Martínez^46^	2.92 (1.68–5.08)	3.81 (1.08–13.45)	72%	0.614

The only confirmatory quantitative meta-analyses (k = 2 each; same exposure and same cancer site, independent samples). The obesity-endometrial estimate is fragile (rests partly on a 66-participant pilot; without it k = 1). OR, odds ratio; CI, confidence interval; FE, fixed-effects; RE, random-effects; I^2^, heterogeneity; τ^2^, between-study variance.

**Table 3:** Direction-of-effect synthesis by behavioral domain

Domain	k	Samples	Protective (OR<1)	Risk (OR>1)	Null (OR = 1)	Median OR	OR range	Binomial p (direction)
Adiposity	6	6	1	5	0	2.40	0.604–8.1	0.219
Alcohol consumption	5	5	0	5	0	2.93	1.16–4.62	0.0625
Cigarette smoking	7	5	0	6	1	4.00	1.00–10.5	0.0312
Healthy diet	9	9	9	0	0	0.41	0.05–0.64	0.00391
Physical activity	3	3	3	0	0	0.61	0.47–0.83	0.250
Unhealthy diet	16	15	1	15	0	1.60	0.6–5.49	0.000519

Synthesis Without Meta-analysis (SWiM) by direction of effect (Campbell et al.). Each estimate is classified by the direction of its point estimate: OR<1 protective, OR>1 risk, OR = 1 exactly null/tie. The two-sided exact binomial test assesses departure from a 1:1 split of directions, with ties excluded (cigarette smoking includes one estimate at OR = 1.00). Median OR and OR range are descriptive only. k = number of estimates; several studies contribute estimates to more than one domain or cancer site; “Samples” is the number of independent samples. Binomial probabilities treat each estimate as the unit; in two domains the number of estimates exceeds the number of independent samples (cigarette smoking, 7 estimates/5 samples; unhealthy diet, 16/15), so these *p* values are approximate and should be read as descriptive rather than confirmatory.

**Table 4:** GRADE summary of findings

Outcome	No. studies/samples	Effect estimate (audited data only)	Certainty (GRADE)	Footnotes (rationale for rating)
DII/E-DII × prostate cancer	2 studies/2 independent samples	Random-effects OR 1.38 (1.11–1.73); fixed-effect OR 1.41 (1.20–1.66); I^2^ = 36%	Low	Start observational (low). RoB: moderate (−0). Inconsistency: I^2^ = 36%, consistent direction (−0). Indirectness: DII vs. energy-adjusted E-DII scales differ (−1). Imprecision: only k = 2 (−1). Publication bias: not assessable, k < 10 (suspected). UPGRADE: positive dose-response across tertile rank (+1); E value supports robustness. Net = low.
Obesity (BMI ≥30) × endometrial cancer	2 studies/2 independent samples	Random-effects OR 3.81 (1.08–13.45); fixed-effect OR 2.92 (1.68–5.08); I^2^ = 72%	Very low	Start observational (low). RoB: one pilot at high risk (Ortiz, 22/44) (−1). Inconsistency: I^2^ = 72% (−1). Imprecision: random-effects CI 1.08–13.45, pilot *n* = 66 (−1). UPGRADE: large effect (OR up to 8.1) (+1). Net = very low. Fragile: without the pilot, k = 1.
Healthy dietary domain (overall direction)	9 estimates/∼9 independent samples	Vote-counting: 9/9 protective; median OR 0.41 (range 0.05–0.64); binomial *p* ≈ 0.004	Low	Directional synthesis (not pooled). Direction highly consistent (no downgrade). Indirectness: heterogeneous constructs (patterns, nutrients, foods) and sites combined (−1). RoB: moderate (−0). Certainty applies to DIRECTION, not magnitude. Net = low (direction); magnitude = very low.
Unhealthy dietary domain (overall direction)	16 estimates	Vote-counting: 15 risk/1 protective/0 null; median OR 1.6 (0.6–5.5)	Very low	Directional synthesis. Inconsistency: consistent direction (15/16 increased risk) but heterogeneous magnitudes, high I^2^ if pooled (−1). Indirectness: heterogeneous constructs/sites (−1). Imprecision: several wide CIs (−1). Net = very low.
Physical activity domain (overall direction)	3 estimates	Vote-counting: 3 protective/0 null; median OR 0.61 (0.47–0.83)	Very low	Directional synthesis. Imprecision: only k = 3, mixed contrasts (−1). Indirectness: different PA definitions/sites (−1). RoB: self-reported PA, recall (−1 partial). Net = very low (suggestive protective direction).
Cigarette smoking domain (overall direction)	7 estimates	Vote-counting: 6 risk/1 null; median OR 4.0 (1.0–10.5)	Low	Directional synthesis. Direction consistent and strong (median OR 4.0). UPGRADE: large effect (+1) and known dose-response/plausibility, offsetting indirectness across sites. Net = low (direction); the single null is cervical (Herrero).
Alcohol consumption domain (overall direction)	5 estimates	Vote-counting: 5 risk/0 null; median OR 2.93 (1.16–4.62)	Very low	Directional synthesis. Inconsistency: risk concentrated in UADT/esophagus, non-significant for breast and colorectal (−1). Indirectness: heterogeneous sites/contrasts (−1). Imprecision (−0/−1). Net = very low/low (site-dependent).
Adiposity domain (overall direction)	6 estimates	Vote-counting: 5 risk/1 protective/0 null; median OR 2.4 (0.60–8.1)	Very low	Directional synthesis. Inconsistency: the single inverse estimate is a premenopausal breast stratum (−1). Indirectness: BMI cut-offs differ (≥25 vs. ≥ 30), sites differ (−1). Reverse-causality plausible (anthropometry measured around diagnosis) (−1). Net = very low.

GRADE certainty applies to the body of evidence for each outcome. For directional (SWiM) rows, certainty refers to the DIRECTION of effect, not its magnitude. RoB, risk of bias; I^2^, heterogeneity; CI, confidence interval; DII/E-DII, (energy-adjusted) dietary inflammatory index; PA, physical activity.

#### Unhealthy diet

**Figure 3:**
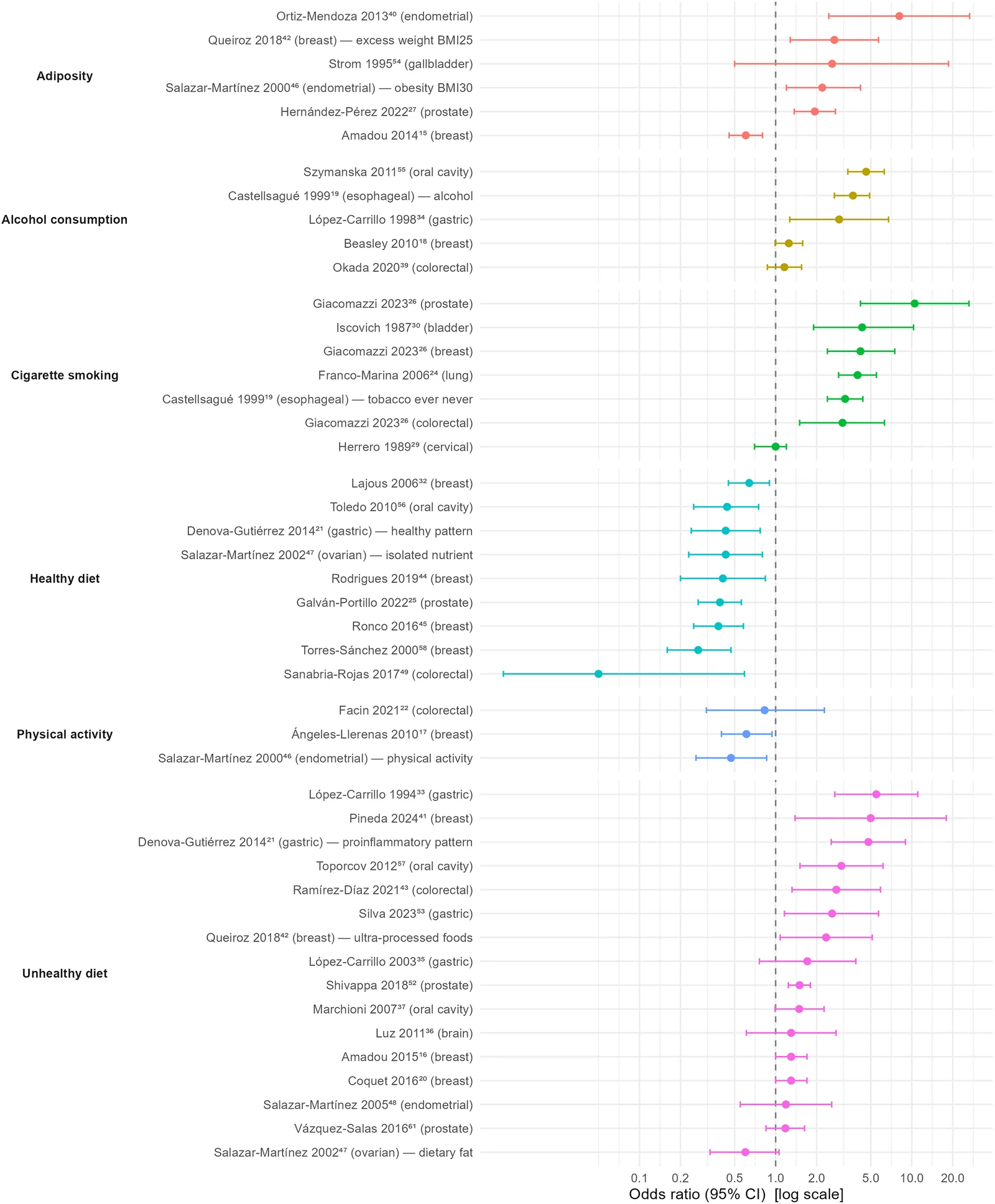
Direction-of-effect forest plots by behavioral domain (descriptive synthesis without meta-analysis; no pooled cross-site estimate) Each figure displays the study-level odds ratios and the domain median for unhealthy diet, tobacco smoking, healthy diet, adiposity, alcohol consumption, and physical activity. Error bars represent 95% confidence intervals

Unhealthy dietary exposures were predominantly associated with increased cancer risk in a direction-of-effect synthesis (15 of 16 estimates indicated increased risk; median OR 1.60; binomial *p* = 0.0005; Table 3; Figure 3). We did not retain a single pooled estimate for unhealthy diet and gastric cancer: the contributing studies measured non-comparable constructs (a refined/Western dietary pattern, chili/capsaicin intake, and the energy-adjusted dietary inflammatory index), and high heterogeneity (I^2^ = 91.7%) reflected this mixture rather than sampling error. On re-verification against the primary source, the most extreme consumption category reported by López-Carrillo (OR 17.11) was an idiosyncratic contrast; under our pre-declared selection rule we used the standardized any-versus-none contrast reported in the same study (OR 5.49, 95% CI 2.72–11.06; Table 1). The previously reported gastric pooled estimate was therefore not retained.

#### Obesity and protective factors

**Figure 4:**
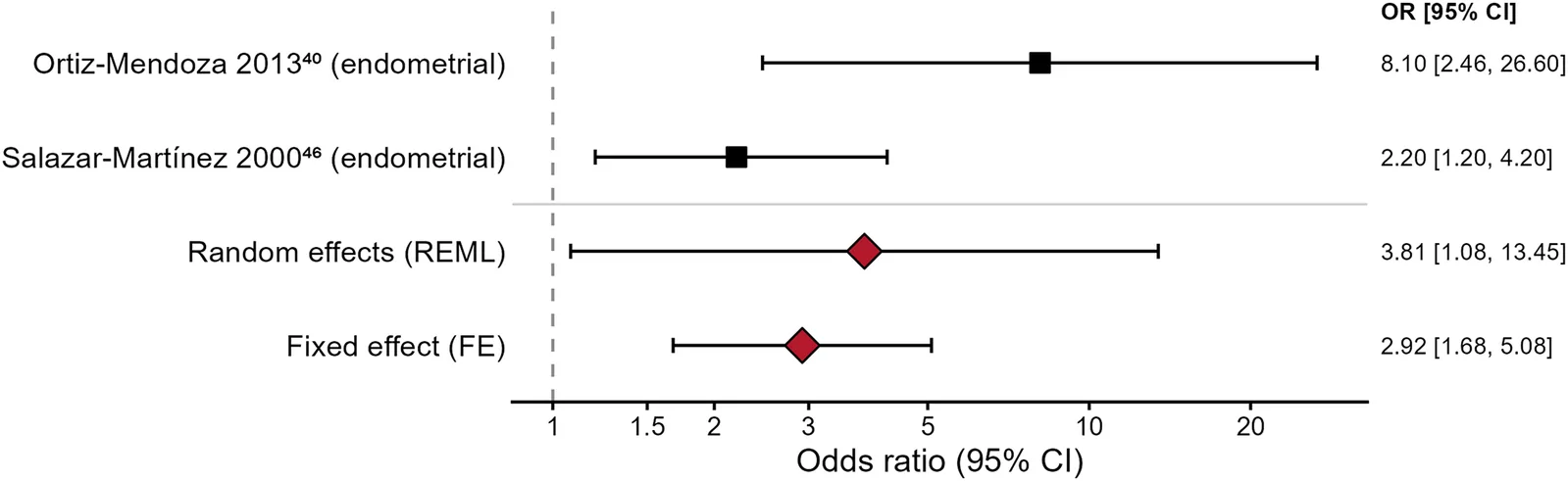
Forest plot of the site-specific pool of obesity and endometrial cancer (random-effects; k = 2) Interpret with caution: the estimate is fragile and rests partly on a 66-participant pilot study, without which only a single study remains. Error bars represent 95% confidence intervals.

Obesity was associated with endometrial cancer in one of only two site-specific pools that met our criteria (two studies; random-effects OR 3.81, 95% CI 1.08–13.45; I^2^ = 72%; Figure 4). This estimate must be interpreted with extreme caution because it rests partly on a 66-participant pilot study, without which only a single study remains. Obesity and breast cancer were not pooled: the contributing estimates pointed in opposite directions (an inverse association in the premenopausal CAMA report using BMI ≥30, where reverse causation is plausible, versus increased risk in a study using BMI ≥25), and they are summarized narratively and by menopausal status (Tables 1 and 3). Healthy dietary exposures were associated with lower cancer risk in every contributing estimate (9 of 9 protective across six cancer sites; median OR 0.41; binomial *p* = 0.0039; Table 3; Figure 2); within breast cancer the four contributing estimates each evaluated a different exposure (phytoestrogens, folate, dietary antioxidants, and vitamin A) and were therefore summarized by direction rather than pooled.

#### Dietary inflammation and prostate cancer

**Figure 5:**
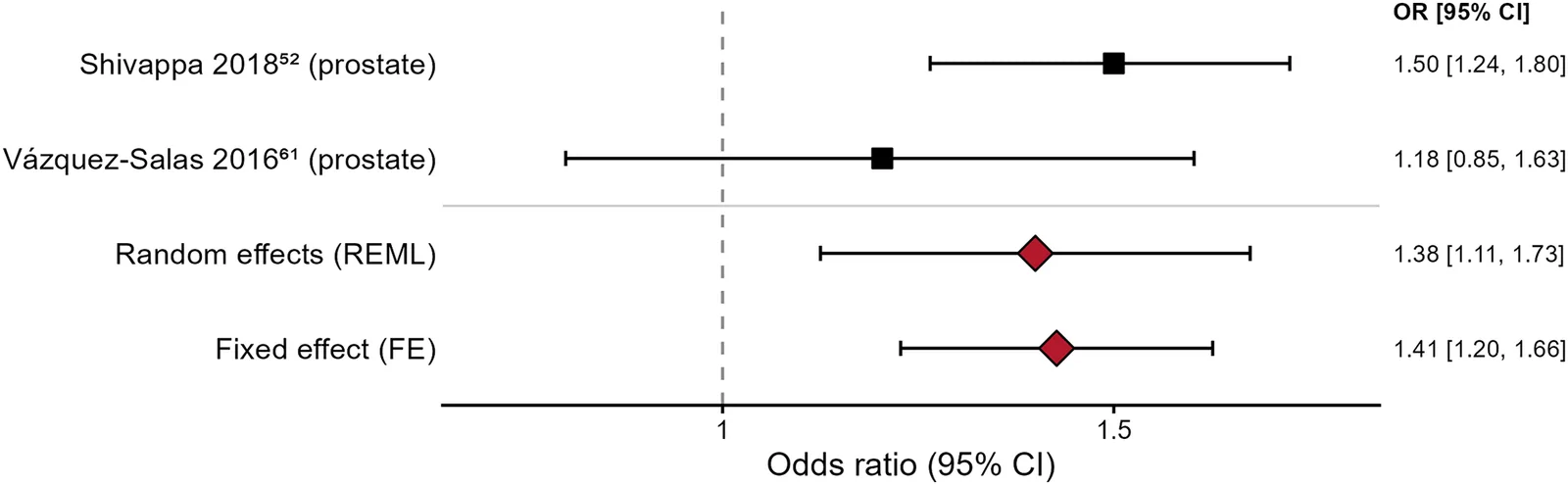
Forest plot of the site-specific pool of the dietary inflammatory index and prostate cancer (random-effects; k = 2; two independent samples sharing a homogeneous exposure construct) Error bars represent 95% confidence intervals.

The dietary inflammatory index and prostate cancer formed the second site-specific pool meeting our criteria (two independent samples, homogeneous construct): a higher index was associated with increased prostate cancer risk (random-effects OR 1.38, 95% CI 1.11–1.73; I^2^ = 36%; Table 2; Figure 5). Because this pool is likewise based on only two studies (k = 2), it must be interpreted with caution. A complementary dose-response analysis (tertile rank) is reported in Table S3 and Figure S3, and its consistent gradient offers partial corroboration.

#### Smoking

Tobacco smoking was consistently associated with increased cancer risk in the direction-of-effect synthesis (6 of 7 estimates indicated increased risk; median OR 4.00; binomial *p* = 0.0312; Table 3; Figure 3). We did not report site-specific smoking pools: after collapsing the male and female strata of the single IARC multicenter study into one estimate (to respect sample independence), the smoking-esophageal contrast was based on a single study and is reported narratively (Table 1). Earlier estimates that treated sex strata as independent studies were therefore not retained.

Associations with insufficient data for pooling (k = 1) are detailed in Table 1.

Assessment of small-study effects was restricted to the global aggregated dataset presented in the supplementary material; this decision was based on the requirement of a minimum of ten studies (k ≥ 10) per meta-analysis for reliable asymmetry testing, a threshold not met by our site-specific subgroups. In this exploratory global analysis, Egger’s tests suggested potential asymmetry for risk factors (*p* < 0.05), likely driven by small-study effects, whereas no significant asymmetry was detected for protective factors.

## Discussion

This systematic review with meta-analysis synthesizes associations between lifestyle risk factors and cancer risk in Latin America. Quantitative pooling was possible for only two site-specific contrasts (the dietary inflammatory index with prostate cancer, and obesity with endometrial cancer); the remaining evidence was summarized by direction of effect. In that synthesis, tobacco smoking and unhealthy dietary exposures were predominantly associated with increased risk, healthy dietary exposures were consistently associated with lower risk, and obesity was most consistently associated with endometrial cancer. These associations should be read as directional rather than causal, given the predominantly case-control design.

Lifestyle risk factors—including unhealthy dietary patterns, tobacco use, obesity, and physical inactivity—are consistently associated with increased cancer risk.^1^ However, most evidence originates from high-income countries, constraining its external validity for Latin America—a region marked by structural inequities, health-system fragmentation, and context-specific sociocultural determinants that modulate both exposure and vulnerability.^14^^,^^62^ In this context, we provide a rigorous, context-sensitive synthesis of observational studies conducted exclusively in Latin American countries—which, to our knowledge, is the first systematic review and meta-analysis focused solely on this region. Our findings reveal region-specific association patterns that can be obscured in global meta-analyses dominated by data from high-income populations,^1^^,^^62^ and they deliver decision-relevant evidence for settings marked by deep structural inequities, limited access to health-care services, and a high burden of non-communicable diseases.

Tobacco smoking was associated with higher cancer risk, consistent with global evidence.^1^^,^^6^ These findings align with prior evidence^62^ identifying smoking as the leading preventable risk factor for several cancers, particularly of the lung, larynx, esophagus, bladder, and oral cavity.^62^^,^^63^ Attributable fractions in Latin America are likely lower than in high-income settings such as the United States, where smoking accounts for over 20% of incident cancers.^63^ Comparable European analyses also rank smoking as the leading modifiable contributor to cancer burden: regional estimates report substantial smoking-attributable fractions across Europe, and the England burden study confirms tobacco as the top driver among modifiable risks.^2^^,^^6^^,^^62^ In Latin America, attributable fractions are likely lower and more variable across countries, consistent with uneven enforcement of comprehensive tobacco-control policies and differences in cohort smoking histories. Several factors may explain this discrepancy, including lower smoking prevalence in some Latin American countries and scientific limitations related to detection, registration, and attribution of cancers to specific risk factors in the region.^2^^,^^64^^,^^65^ Substantial heterogeneity was evident in the exploratory cross-site overview (Table S2), most plausibly reflecting differences in exposure intensity (e.g., pack years vs. current status), contrasts, and study designs rather than biological inconsistency, since the direction of risk was uniform across the cancer sites analyzed. This variability limits the precision of any cross-site summary and reinforces the need for future studies using standardized designs and harmonized measurement protocols. While smoking prevalence has declined by approximately 1% per year over the past decade, tobacco remains the third most important global risk factor for disability-adjusted life years, after high systolic blood pressure and fasting plasma glucose.^64^ Moreover, more than 60% of adults in countries such as Mexico, Chile, and Brazil live with excess body weight, amplifying the burden of cancer and other non-communicable diseases in these settings.^66^ Collectively, these findings argue for sustained public-health attention to the tobacco-attributable cancer burden and for stronger regulatory policies—particularly in middle-income countries—where substantial gaps persist in tobacco control and primary prevention.

In the identified studies, alcohol consumption was consistently associated with increased cancer risk, particularly for breast, gastric, and upper aerodigestive tract malignancies, consistent with mechanistic evidence identifying ethanol and acetaldehyde as group 1 carcinogens.^11^ A minimum measurement agenda for Latin America should include: reporting ethanol intake in grams/day with explicit beverage-specific alcohol by volume conversion; capturing drinking patterns (regular vs. binge) and beverage type; defining the reference group as lifetime abstainers (separating former drinkers to avoid “sick-quitter” bias); and standardizing a core confounder set (age, sex, tobacco smoking status, socioeconomic status, and—where appropriate—diet quality/total energy intake). Whenever possible, studies should report both continuous contrasts (e.g., per 10 g/day) and categorical dose-response and provide sex-specific estimates to improve comparability and interpretability across Latin American settings. These measurement standards mirror best practices in large European cohorts and high-quality studies—quantifying intake in grams/day, modeling dose-response across cancer sites, and using lifetime abstainers (not former drinkers) as the reference to avoid “sick-quitter” bias—procedures consistently applied in EPIC analyses, meta-analyses, and prevention frameworks.^11^ In addition, recent global and regional assessments reinforce the need for pattern-sensitive metrics (e.g., binge versus regular drinking) and standardized confounder sets to improve comparability across settings.^65^ Because only a few independent studies informed the alcohol domain, these results are exploratory and should be interpreted with caution.

Obesity showed a positive association with cancer risk, particularly for endometrial cancer, consistent with global evidence.^1^^,^^62^^,^^67^ The relationship is plausibly mediated by chronic systemic inflammation, hyperinsulinemia and insulin resistance, hormonal dysregulation, and gut-microbiome perturbations, which collectively shape the tumor microenvironment and promote tumor progression.^68^ In Latin America, obesity prevalence has risen steadily since the 1990s with no clear signs of reversal in most countries.^69^ This epidemiologic backdrop likely contributes to the risk patterns observed in our included studies, particularly given that obesity is a shared risk factor for multiple non-communicable diseases. These findings underscore the need for comprehensive public policies that foster healthy food environments, reduce sedentary behavior, and improve structural conditions that enable active lifestyles. Given likely non-differential misclassification from self-reported measures (which generally biases estimates toward the null), the true association may be underestimated; this reinforces the need for validated instruments and harmonized adjustment sets in Latin American cohorts.

The evidence base is geographically concentrated: 24 of the 47 studies were conducted in Mexico and 10 in Brazil, with the remaining countries contributing one to four studies each. The findings may therefore not generalize to other Latin American countries, whose demographic structure, socioeconomic conditions, health-system organization, and lifestyle profiles differ substantially.

These patterns should be read against the region’s rapid nutrition transition, accelerating urbanization, unequal access to health care, and marked socioeconomic inequality, all of which shape both the distribution of exposures and vulnerability to cancer.

Construct definitions for healthy diet and physical activity varied widely across studies (from single micronutrients to composite dietary patterns; from MET-minutes per week to categorical activity levels), and non-differential exposure misclassification—expected with self-reported instruments—would generally bias associations toward the null. In a sensitivity check restricting the healthy-diet domain to composite dietary patterns (excluding isolated nutrients and single foods), the protective direction was unchanged (4 of 4 versus 5 of 5 estimates protective in the two subsets; Table S2).

### Implications and future directions

Despite its limitations, this meta-analysis delivers a novel quantitative synthesis of associations between lifestyle risk factors and cancer in Latin America—a region historically under-represented in the international literature. By focusing on Latin American settings, it brings to light region-specific patterns and provides prediction intervals that are directly useful for planning in jurisdictions where effect sizes may plausibly be smaller or larger than the pooled estimates.

The findings support the implementation of integrated policies targeting tobacco control, promotion of healthy diets, and prevention of overweight and obesity. While the direction of effects is consistent with the global evidence base, the magnitude and between-study heterogeneity observed here argue for strategies tailored to local realities, including contextual social determinants and varying access to health care. Our findings support the scaling up of successful regional initiatives, such as front-of-package warning labeling in Chile, taxation of sugar-sweetened beverages in Mexico, and robust tobacco control programs in Brazil.^10^^,^^65^

Looking ahead, the limitations identified underscore the need to strengthen regional scientific capacity by developing national cohorts, harmonizing protocols and operational definitions, and increasing investment in analytic and technological infrastructure. Consolidating interoperable data systems (e.g., cancer registries and survey linkages), adopting validated measurement tools, and improving transparent reporting (pre-registration, shared code, and metadata) will be critical to generating context-specific, reproducible evidence that can inform effective public policy across Latin America. Adopting pre-registration, shared code, and standardized reporting (including site-specific definitions and exposure harmonization) will accelerate reproducible, policy-relevant evidence across the region. A composite index of healthy behaviors (for example, Life’s Essential 8) is a promising future direction, but it will require individual-level rather than aggregated study data.

### Limitations of the study

Despite the novelty and relevance of this work, it is not free from limitations inherent to the designs included. First, the predominance of case-control studies (46 of 47; 97.9%) limits causal inference and increases susceptibility to selection and recall bias. Second, substantial heterogeneity (I^2^ > 75%) was evident in some exploratory groupings that mixed non-comparable exposure constructs (e.g., the heterogeneous unhealthy-diet exposures evaluated within gastric cancer) or different cancer sites; because these groupings combined non-comparable constructs, they were not pooled; the evidence is instead summarized by direction of effect, which remained consistent across primary studies (Figure 3). This heterogeneity likely stems from cross-study differences in covariate adjustment and exposure definitions. A major limitation is the lack of harmonization in defining “healthy diet” across Latin American studies, ranging from specific micronutrients (e.g., folate) to composite dietary patterns, which complicates direct comparison. In addition, incomplete reporting restricted uniform sensitivity analyses. The limited number of studies for certain exposures (e.g., physical inactivity and alcohol) precluded robust estimation; specifically, heterogeneity in physical activity definitions (e.g., MET-min/week vs. categorical) further constrained comparability. Small-study effects could not be robustly assessed where k < 10, consistent with our *a priori* analytic plan. Finally, because we selected a single estimate per study, selection bias within studies cannot be ruled out. Reverse causation is additionally plausible for anthropometric exposures, which were typically measured around the time of diagnosis. Formal subgroup analysis and meta-regression were not feasible: only two site-specific pools (each of two studies) were available, far below the approximately ten studies per covariate required for stable meta-regression. Where key data were missing or ambiguous, we attempted to contact the corresponding authors of the primary studies; responses were limited and, for several older studies, the authors could not be reached, so estimates that could not be clarified were handled conservatively (reclassified to narrative synthesis or used only in sensitivity analyses). Finally, most included studies addressed sex-specific cancer sites (breast, prostate, endometrium, and ovary) or reported sex-stratified estimates only, and the aggregate nature of the data precluded a formal assessment of how sex or gender influenced the associations; this could not be examined further and is acknowledged as a limitation.

## Resource availability

### Lead contact

Further information and requests for resources should be directed to and will be fulfilled by the lead contact, Gerson Ferrari (gerson.demoraes@usach.cl).

### Materials availability

This study did not generate new unique reagents.

### Data and code availability

• All data supporting the findings of this study were derived from previously published articles. The study-level extracted data used for the meta-analyses are provided in this article and the supplemental information.
• The meta-analyses were conducted in R using standard functions available in the metafor and clubSandwich packages; no custom code was developed.
• Any additional information required to reanalyze the data reported in this study is available from the lead contact upon request.

## Acknowledgments

The authors thank the Vicerrectoría de Investigación, Desarrollo e Innovación at Universidad de Santiago de Chile (USACH) for their support. This study received financial support from 10.13039/501100024579Vicerrectoría de Investigación, Desarrollo e Innovación, Universidad de Santiago de Chile (USACH), Proyecto DICYT, grant #032504DF_Postdoc. LFMR is supported by the 10.13039/501100003593National Council for Scientific and Technological Development—CNPq (311109/2023-3).

## Author contributions

G.B.-F.: conceptualization, methodology, data curation, formal analysis, writing – original draft, review, and editing; L.F.M.R.: methodology, supervision, and writing – review and editing; J.W.: data curation and writing – review and editing; P.F.-H.: data curation and writing – review and editing; E.N.: validation and writing – review and editing; G.F.: conceptualization, methodology, supervision, and writing – review and editing.

## Declaration of interests

The authors declare no competing interests.

## Declaration of generative AI and AI-assisted technologies in the writing process

During the preparation of this work, the authors used Gemini (Google) in order to improve the readability and language of the manuscript, and to assist in the extraction and cross-checking of effect-size data from the primary studies. All extracted estimates were independently verified by the authors against the original publications (table, page, and reported values), consistent with a human-in-the-loop approach in which AI outputs were treated as provisional and subject to full author verification. The authors additionally used Turnitin to screen the manuscript for textual similarity. After using these tools and services, the authors reviewed and edited the content as needed and take full responsibility for the content of the published article.

## STAR★Methods

### Key resources table

**Table:** 

REAGENT or RESOURCE	SOURCE	IDENTIFIER
**Deposited data**		

Study-level extracted effect-size dataset (audited)	This paper	Supplementary materials
Included primary studies (*n* = 47)	This paper	Reference list, references 15–61 (with DOIs)
Registered review protocol	PROSPERO	CRD420251042385; https://www.crd.york.ac.uk/prospero/

**Software and algorithms**		

R (version 4.4.0)	R Foundation for Statistical Computing	https://www.r-project.org/
R package: metafor	Viechtbauer	https://CRAN.R-project.org/package=metafor
R package: dosresmeta	Crippa & Orsini	https://CRAN.R-project.org/package=dosresmeta
R package: clubSandwich	Pustejovsky	https://CRAN.R-project.org/package=clubSandwich
R package: tidyverse	Wickham et al.	https://CRAN.R-project.org/package=tidyverse
Microsoft Excel	Microsoft	https://www.microsoft.com/en-us/microsoft-365/excel
QUIPS (Quality In Prognosis Studies) risk-of-bias tool	Hayden et al.	N/A

**Other**		

Data published/compiled from the literature	Web of Science	https://webofscience.com
Data published/compiled from the literature	Scopus	https://www.scopus.com
Data published/compiled from the literature	MEDLINE/PubMed	https://pubmed.ncbi.nlm.nih.gov/
Data published/compiled from the literature	LILACS	https://lilacs.bvsalud.org/

### Method details

#### Study design and framework

This systematic review and meta-analysis was conducted in accordance with the PRISMA 2020 and MOOSE guidelines,^70^^,^^71^ and was prospectively registered in PROSPERO (CRD420251042385). We framed the research question using the PECO(S) structure—Population, Exposure, Comparator, Outcome (and Study design/Setting)—as recommended for reviews of etiologic associations in the JBI Manual for Evidence Synthesis (Etiology and Risk) and the Cochrane Handbook. This framework is appropriate for observational studies relating modifiable exposures to cancer incidence, accommodates case–control and cohort designs, and facilitates transparent specification of contrasts, outcomes, and contextual features relevant to Latin America.

#### Eligibility criteria

We included analytical observational studies—prospective/retrospective cohorts and case–control designs—recruiting adults (≥18 years) residing in Latin American countries (P); multicountry reports were eligible only when Latin American-stratified estimates were available. The exposures were tobacco smoking, alcohol consumption, adiposity (overweight/obesity or related metabolic indicators), physical inactivity/sedentary behavior, and dietary patterns (healthy/unhealthy); exposure had to be ascertained for a pre-diagnostic period (cohorts: before incidence; case–control: with a clearly referenced pre-diagnosis window). We included studies with validated self-report or objective measures and recorded instrument type and scaling for harmonization (E). The comparator was lower or no exposure (e.g., never smokers, lifetime abstainers for alcohol, lowest quantile for diet/adiposity/physical activity) or per-unit increases for continuous contrasts; the original reference/contrast was extracted verbatim and later harmonized (C). The outcome was incident primary cancer (all sites or site-specific) confirmed by histology, cancer registries, or ICD codes; we excluded studies focused solely on mortality, survival, progression, recurrence, precancerous lesions, or molecular biomarkers without incident cancer (O). The setting was restricted to Latin America due to shared epidemiologic burden and socio-structural patterns; we excluded cross-sectional and ecological designs (S). Eligible studies reported OR, RR, or HR with 95% CIs, or provided sufficient data to compute them; when multiple reports overlapped, we retained the most complete, non-overlapping dataset. No language or date restrictions were applied.

#### Search strategy and selection criteria

The literature search was conducted in Web of Science, Scopus, MEDLINE, and LILACS, from each database’s inception to September 2025, without language restrictions. Controlled vocabulary and free-text terms were combined using Boolean operators; [(“cancer” OR “neoplasms”) AND (“modifiable risk factors” OR “lifestyle factors” OR “smoking” OR “tobacco” OR “alcohol consumption” OR “obesity” OR “physical inactivity” OR “diet”) AND (“cohort study” OR “cohort studies” OR “case-control study” OR “case control studies”) AND (“Latin America” OR “Central America” OR “South America” OR “Caribbean Region” OR “Mexico”)]. Two independent reviewers (G.B-F and G.F) screened titles/abstracts and full texts. Discrepancies were resolved through consensus. The complete search syntax adapted to each database (PubMed/MEDLINE, Scopus, Web of Science, and LILACS), with field tags and Boolean operators, is provided in the Supplementary Search Strategy.

#### Data extraction and processing

Data were systematically recorded in a Microsoft Excel spreadsheet developed by the research team. For each study, we extracted: author, year, country, study design, type of exposure, type of cancer, effect estimate (OR), 95% confidence interval (lower and upper bounds), sample size, number of cases and controls (for case-control studies) or participants (cohort studies), whether multivariable adjustment was performed (yes/no), covariates used in adjustment. We pre-specified a hierarchical selection rule prioritizing (i) fully-adjusted over crude estimates; (ii) standardized exposure contrasts over idiosyncratic categorizations; and (iii) population-based over convenience samples. Three sensitivity analyses were performed: an alternative exposure contrast for the gastric outlier (any-versus-none versus the most extreme category for López-Carrillo), a restriction of the healthy-diet domain to composite dietary patterns versus isolated nutrients (Table S2), and a restriction of the direction-of-effect synthesis to low-risk-of-bias studies (Table S5).

Direction of effects and contrast harmonization: To ensure semantic coherence across domains, contrasts were harmonized before pooling. For protective domains (healthy diet and physical activity), higher exposure denotes healthier behavior; when primary studies reported the reverse orientation (e.g., higher scores = unhealthier diet; low vs. high activity as reference), we inverted the contrast so that pooled ORs < 1 consistently indicate protection. For harmful domains (tobacco smoking, alcohol consumption, obesity), higher exposure denotes higher risk, and pooled ORs > 1 indicate harm. Inversions were performed on the log scale—i.e., log(OR_inv) = −log(OR), with the 95% CI obtained by inverting the original bounds (1/upper, 1/lower)—and verified for consistency with the study’s coding. Where studies used non-comparable contrasts (e.g., per-SD vs. quantiles), the original contrast was retained and contrast type was recorded *a priori* as a potential moderator for meta-regression.

#### Risk of bias assessment

Methodological quality was appraised with the Quality in Prognosis Studies (QUIPS) tool, recommended for reviews of prognostic or exposure factors. The unit of assessment was each study–outcome pair. Two reviewers (G.B-F and G.F), after a calibration exercise, independently rated all studies; disagreements were resolved by consensus with a third reviewer available. Each domain was judged low, moderate, or high risk of bias using prespecified operational criteria tailored to case–control and cohort designs. *Study participation (selection)* evaluated representativeness and comparability of the source population (population- or community-based sampling and controls from the same catchment favored low risk; clearly selective or non-comparable controls indicated higher risk). *Study attrition* was interpreted as participation/non-response in case–control studies and loss to follow-up in cohorts; clearly reported, similar rates implied low risk, whereas sparse reporting or marked differential loss suggested higher risk. *Prognostic factor (exposure) measurement* assessed validity, standardization, and timing; validated instruments or objective measures (e.g., trained anthropometry, biomarkers) counted as low risk, whereas self-report without validation was considered moderate and evidence of differential misclassification high. Given the topic, FFQ-based dietary exposures were classified as moderate by default and could be upgraded to low only when prior validation and standardized administration were documented. *Outcome measurement* prioritized objective ascertainment (histology, cancer registries, ICD codes) as low risk; self-report without verification was high. *Confounding* judged identification and control of *a priori* confounders appropriate to each exposure–outcome pair, evaluated against a predefined list of domain-specific covariates (detailed in Table 1); low risk required matching and/or multivariable adjustment for key factors (e.g., age, sex, education/SES, smoking, total energy for dietary exposures, BMI/anthropometry, relevant family history), while crude estimates or omission of obvious confounders were high. *Statistical analysis and reporting* appraised model appropriateness and transparency (e.g., logistic or Cox models, OR with 95% CIs, handling of exposure scaling/trends, checks of assumptions, and missing-data strategies). For operational synthesis we computed an overall risk of bias by two prespecified rules: the primary rule classified studies as high if any domain was high, moderate if ≥2 domains were moderate (and none high), and low if ≤ 1 domain was moderate (and none high) (Table 1). Effect-size extraction was supported by AI-assisted cross-checking, but every extracted estimate was independently verified by the authors against the original publication (table, page, and reported values); the QUIPS risk-of-bias assessment and all synthesis decisions were made by the authors and were not delegated to AI.

### Quantification and statistical analysis

To account for biological heterogeneity across cancer sites, we prioritized site-specific meta-analyses where sufficient data were available (k ≥ 2). Global pooled estimates (across all sites) are provided in the Supplementary Material solely to offer a regional burden overview, utilizing a multilevel random-effects model (rma.mv) nested by study ID to account for correlated estimates within the same study population. After the harmonization described above, each effect estimate was transformed to its natural logarithm—log(OR), log(RR), or log(HR)—and standard errors (SE) were computed using SE = [log(upper CI) − log(lower CI)] / (2 × 1·96). Random-effects models were fitted using restricted maximum likelihood (REML); where applicable, Knapp–Hartung adjustments were used for test statistics and confidence intervals. We reported pooled estimates with 95% confidence intervals, heterogeneity metrics (I^2^, τ^2^, Cochran’s Q), and 95% prediction intervals (reported for k ≥ 3). Forest plots were generated for each risk factor stratified by cancer site. Meta-regression by study-level moderators was pre-specified but proved infeasible, because each poolable set contained only two studies (far below the ≈10 studies per covariate required for stable meta-regression). Small-study effects were assessed using Egger’s regression and Begg’s rank correlation only when k ≥ 10; these tests were not conducted or reported below that threshold. Quantitative pooling was restricted to site-specific sets of two or more independent samples (k ≥ 2) that evaluated the same exposure contrast in the same cancer site; estimates sharing a study population were nested by a sample identifier and collapsed before pooling so that no participant contributed more than once. Because only a small number of site-specific sets were poolable, the remaining evidence was summarized using a structured direction-of-effect synthesis (Synthesis Without Meta-analysis, SWiM): for each behavioral domain we tabulated the number of estimates indicating decreased versus increased risk, the median odds ratio, and a two-sided exact binomial probability under the null of equal direction. The full body of evidence was displayed in an albatross plot, which relates study *p*-values to sample size on a contour of effect size. As an exploratory sensitivity analysis only, domain-level estimates were additionally computed across cancer sites using random-effects and three-level models; because these combine heterogeneous exposures and different cancer sites they are not site-specific, carry no causal interpretation, and are reported only in the Supplement. For domains with fewer than five independent clusters (physical activity, alcohol) the robust confidence interval is not interpretable and is not reported. For all included estimates we computed E-values (Table S4; Figure S4) to quantify the minimum strength of unmeasured confounding that could explain away the association. Where tertile-level data were available (dietary inflammatory index and prostate cancer), a two-stage dose–response meta-analysis was fitted using tertile rank as the dose metric. The certainty of evidence was summarized with the GRADE approach in a Summary-of-Findings table (Table 4).

#### Statistical software and reproducibility

All analyses were conducted in R version 4.4.0 (Windows), using reproducible scripts in RMarkdown. We used the metafor, dosresmeta, clubSandwich, tidyverse, writexl, officer, flextable, and knitr packages. Forest plots were exported as high-resolution images and included in the final report. Summary tables include results by exposure group and a detailed supplementary table with data per study (exposure, outcome, design, estimate, sample size, country, adjustment, and period).

### Additional resources

The protocol was prospectively registered with PROSPERO (CRD420251042385; https://www.crd.york.ac.uk/PROSPERO/).
