# Supplementary material for: Modifiable lifestyle factors and cancer risk in Latin America: A systematic review with meta-analysis
Source: iScience. 2026 Sep 5;29(9):117381. doi: 10.1016/j.isci.2026.117381 (PMC13571283; doi:10.1016/j.isci.2026.117381)
Supplement: Supplementary file 1 [file mmc1.pdf]

iScience, Volume *29*

Supplemental information

Modifiable lifestyle factors and cancer risk in Latin America: A systematic review with meta-analysis

Guillermo Barahona-Fuentes, Leandro F.M. Rezende, Jacqueline Wahrhaftig, Paloma Ferrero-Hernández, Eduardo Nilson, and Gerson Ferrari

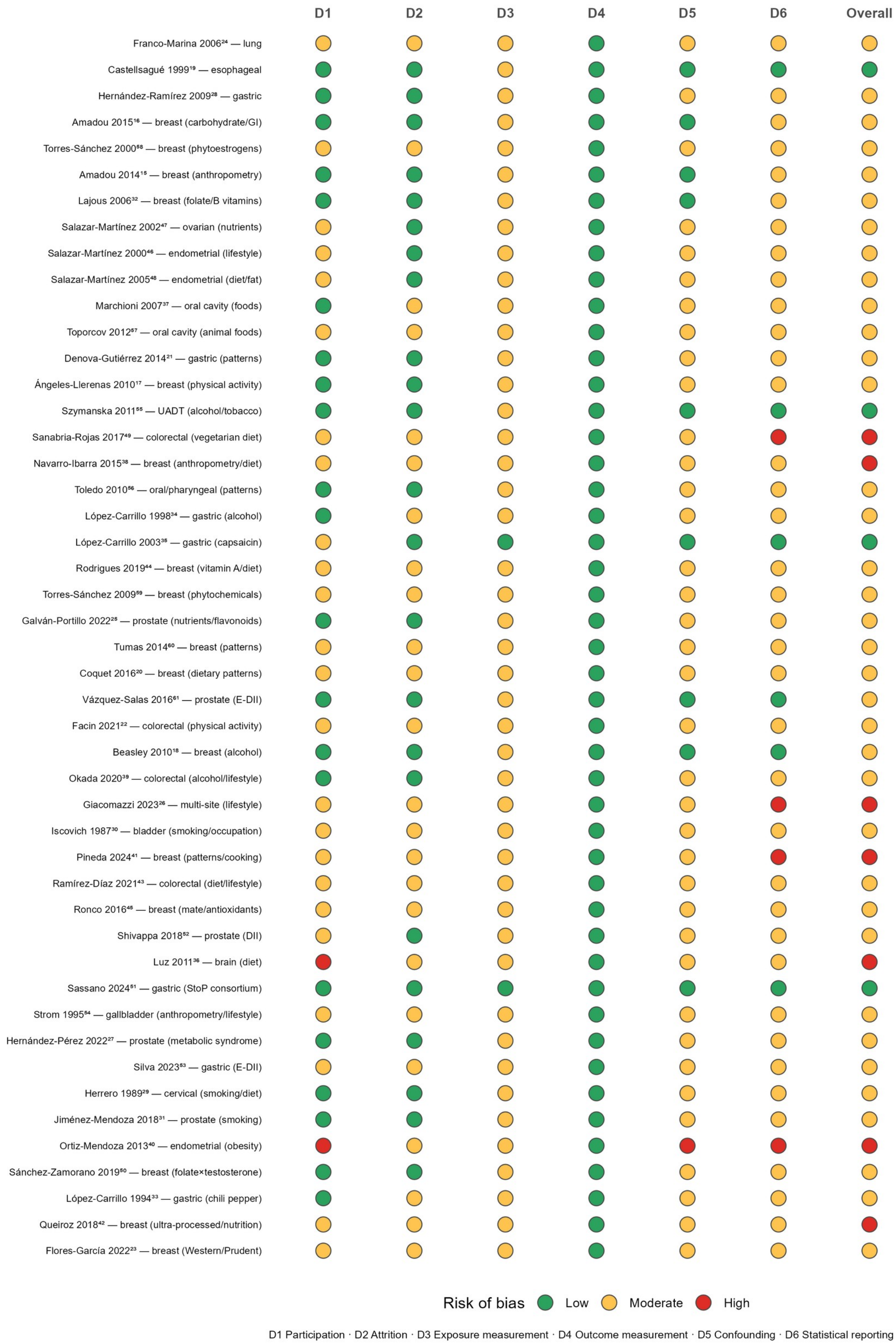

D1
D2
D3
D4
D5
D6
Overall
Franco-Marina 2006[24] — lung
Castellsagué 1999[19] — esophageal
Hernández-Ramírez 2009[28] — gastric
Amadou 2015[16] — breast (carbohydrate/GI)
Torres-Sánchez 2000[58] — breast (phytoestrogens)
Amadou 2014[15] — breast (anthropometry)
Lajous 2006[32] — breast (folate/B vitamins)
Salazar-Martínez 2002[47] — ovarian (nutrients)
Salazar-Martínez 2000[46] — endometrial (lifestyle)
Salazar-Martínez 2005[48] — endometrial (diet/fat)
Marchioni 2007[37] — oral cavity (foods)
Toporcov 2012[57] — oral cavity (animal foods)
Denova-Gutiérrez 2014[21] — gastric (patterns)
Ángeles-Llerenas 2010[17] — breast (physical activity)
Szymanska 2011[55] — UADT (alcohol/tobacco)
Sanabria-Rojas 2017[49] — colorectal (vegetarian diet)
Navarro-Ibarra 2015[38] — breast (anthropometry/diet)
Toledo 2010[56] — oral/pharyngeal (patterns)
López-Carrillo 1998[34] — gastric (alcohol)
López-Carrillo 2003[35] — gastric (capsaicin)
Rodrigues 2019[44] — breast (vitamin A/diet)
Torres-Sánchez 2009[59] — breast (phytochemicals)
Galván-Portillo 2022[25] — prostate (nutrients/flavonoids)
Tumas 2014[60] — breast (patterns)
Coquet 2016[20] — breast (dietary patterns)
Vázquez-Salas 2016[61] — prostate (E-DII)
Facin 2021[22] — colorectal (physical activity)
Beasley 2010[18] — breast (alcohol)
Okada 2020[39] — colorectal (alcohol/lifestyle)
Giacomazzi 2023[26] — multi-site (lifestyle)
Iscovich 1987[30] — bladder (smoking/occupation)
Pineda 2024[41] — breast (patterns/cooking)
Ramírez-Díaz 2021[43] — colorectal (diet/lifestyle)
Ronco 2016[45] — breast (mate/antioxidants)
Shivappa 2018[52] — prostate (DII)
Luz 2011[36] — brain (diet)
Sassano 2024[51] — gastric (StoP consortium)
Strom 1995[54] — gallbladder (anthropometry/lifestyle)
Hernández-Pérez 2022[27] — prostate (metabolic syndrome)
Silva 2023[53] — gastric (E-DII)
Herrero 1989[29] — cervical (smoking/diet)
Jiménez-Mendoza 2018[31] — prostate (smoking)
Ortiz-Mendoza 2013[40] — endometrial (obesity)
Sánchez-Zamorano 2019[50] — breast (folate×testosterone)
López-Carrillo 1994[33] — gastric (chili pepper)
Queiroz 2018[42] — breast (ultra-processed/nutrition)
Flores-García 2022[23] — breast (Western/Prudent)
Risk of bias
Low
Moderate
High
D1 Participation · D2 Attrition · D3 Exposure measurement · D4 Outcome measurement · D5 Confounding · D6 Statistical reporting

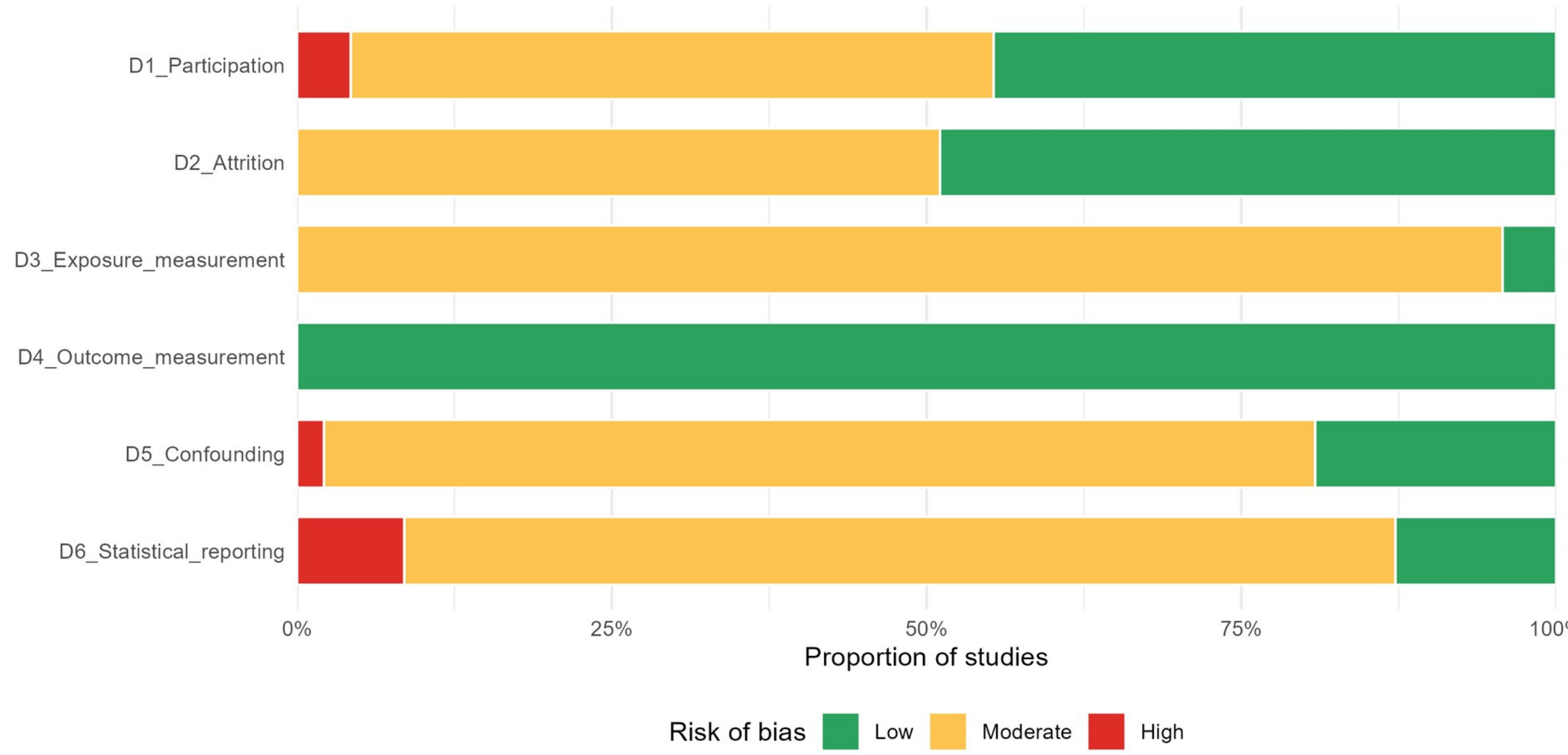

Figure S1. Risk-of-bias assessment using the QUIPS tool. (a) Traffic-light plot showing the rating for each of the six domains and the overall judgement for every included study. (b) Summary bar plot showing the distribution of domain-level ratings across studies. D1, study participation; D2, study attrition; D3, prognostic-factor (exposure) measurement; D4, outcome measurement; D5, confounding; D6, statistical analysis and reporting.

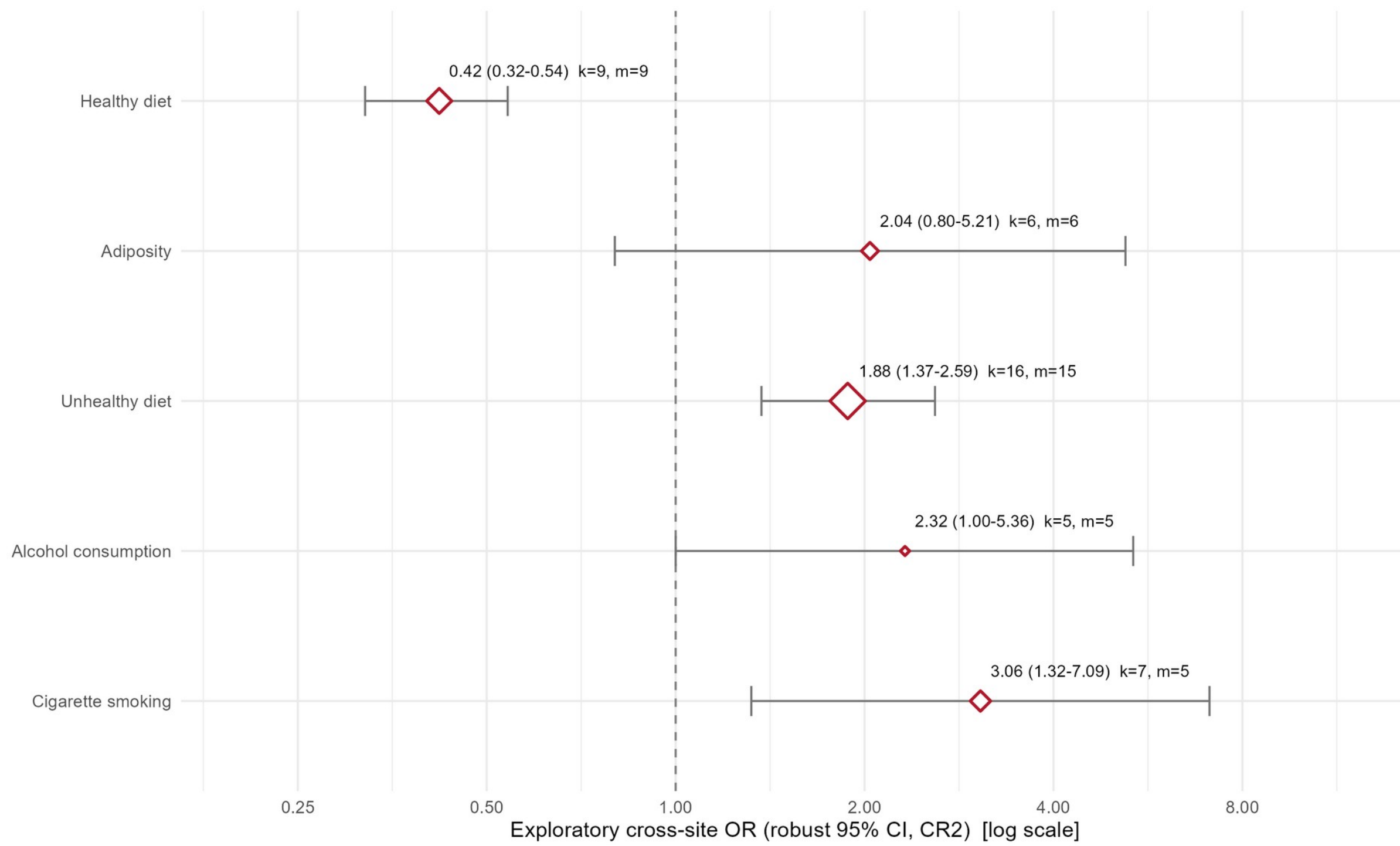

Figure S2. Exploratory domain-level synthesis across cancer sites (supplementary; not site-specific and not confirmatory). Heterogeneous exposures and different cancer sites are combined; domains with fewer than five clusters are omitted. Estimates are pooled odds ratios from three-level random-effects models with cluster-robust (CR2) 95% confidence intervals; k, number of estimates; m, number of independent samples.

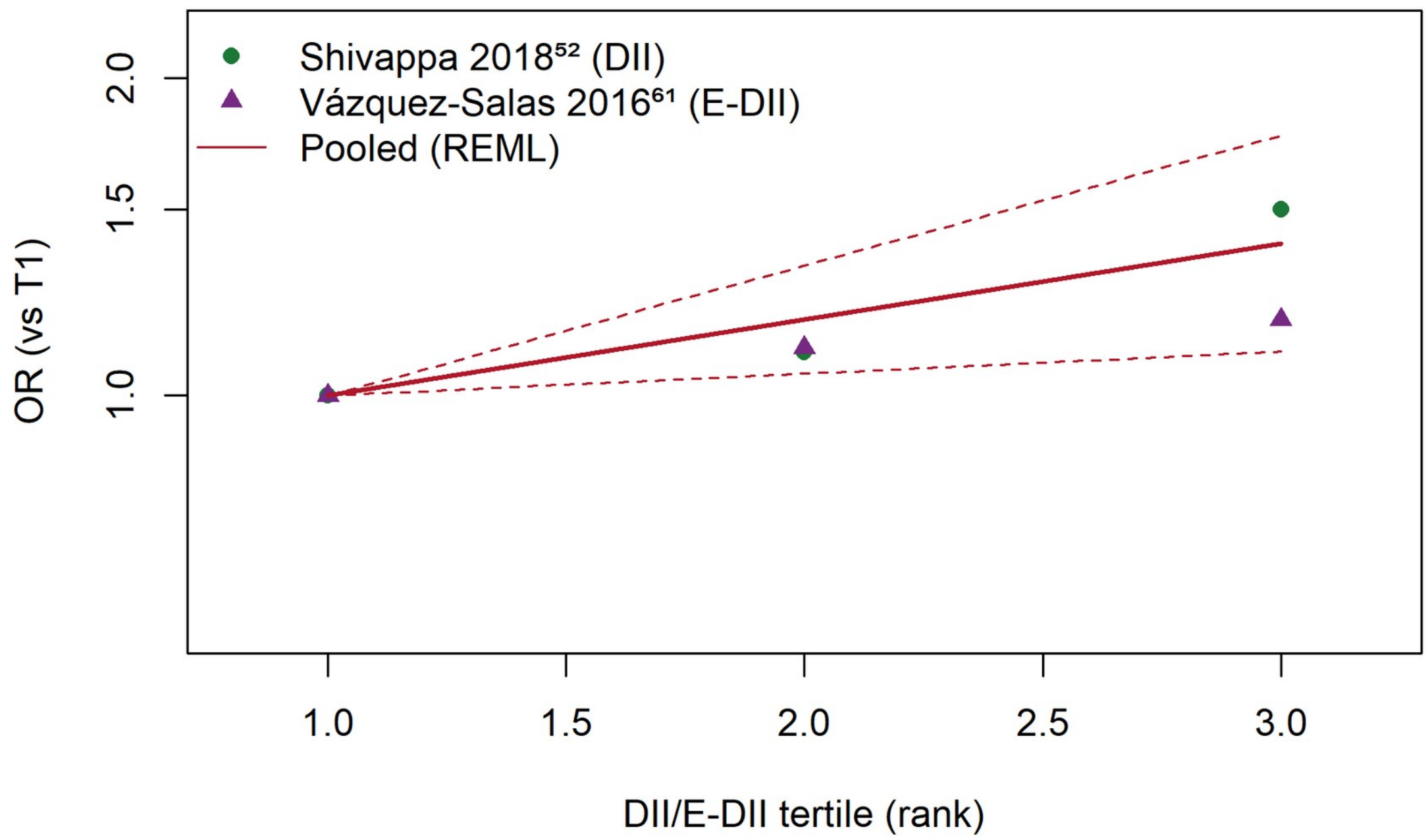

Figure S3. Dose–response relationship between the dietary inflammatory index (tertile rank) and prostate cancer. Two-stage (Greenland–Longnecker) dose–response model fitted by restricted maximum likelihood; the solid line is the pooled trend and dashed lines are 95% confidence limits.

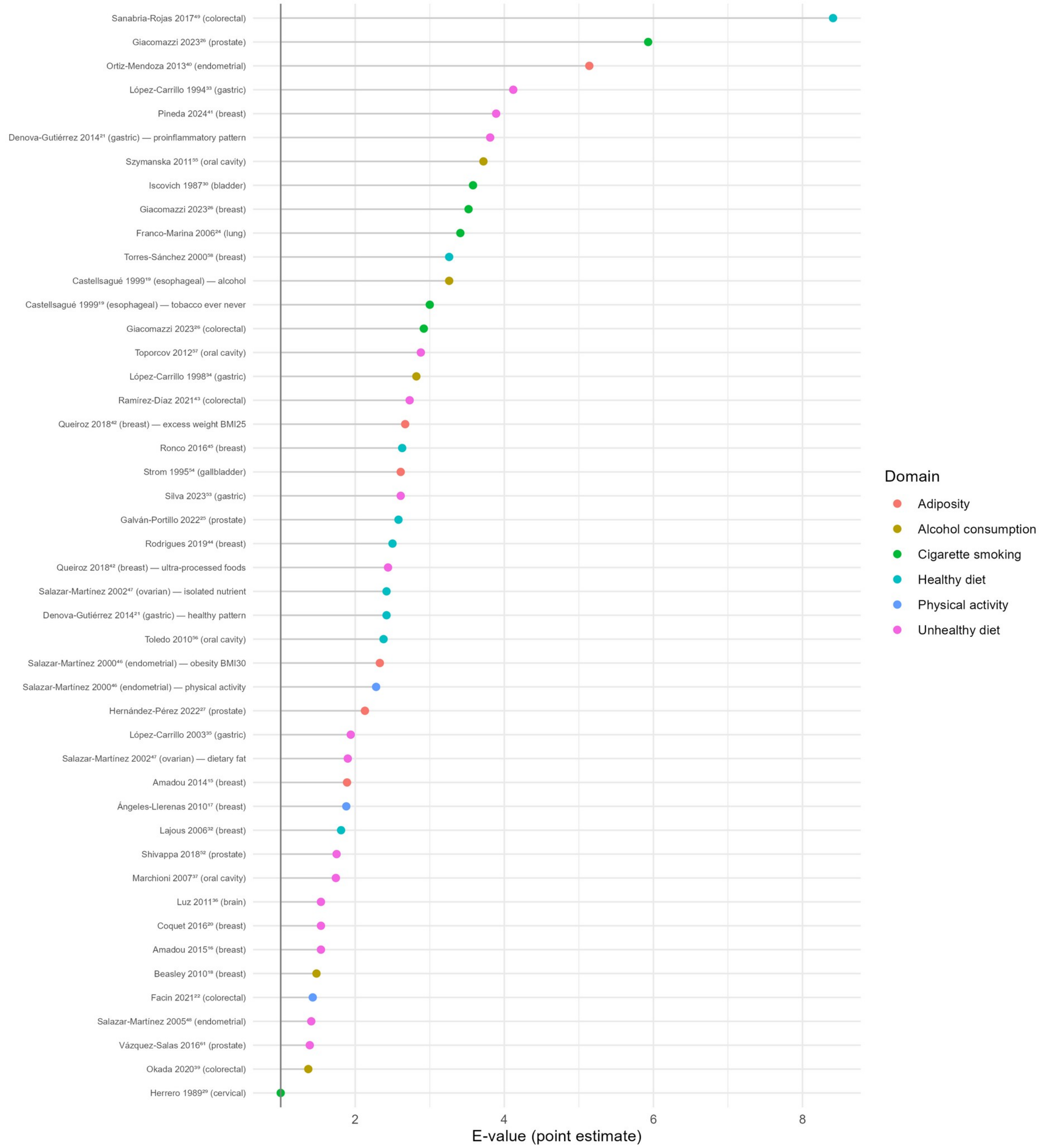

Figure S4. E-values for each site-specific and domain-level association, ordered by magnitude. The E-value is the minimum strength of association, on the risk-ratio scale, that an unmeasured confounder would need with both the exposure and the outcome to fully explain away the observed estimate; larger values indicate greater robustness to unmeasured confounding.

Supplementary Table S1. Risk-of-bias assessment (QUIPS, six domains)

Authors	Study participation	Study attrition	Prognostic factor measurement	Outcome measurement	Confounding	Statistical analysis & reporting	Overall risk of bias
Amadou et al., 2014 [15]	Low	Low	Moderate	Low	Low	Low	Low
Amadou et al., 2015 [16]	Low	Low	Moderate	Low	Low	Low	Low
Ángeles-Llerenas et al., 2010 [17]	Low	Low	Moderate	Low	Low	Low	Low
Beasley et al., 2010 [18]	Low	Low	Moderate	Low	Low	Low	Low
Castellsagué et al., 1999 [19]	Moderate	Low	Moderate	Low	Low	Low	Moderate
Coquet et al., 2016 [20]	Low	Moderate	Moderate	Low	Low	Low	Moderate
Denova-Gutiérrez et al., 2014 [21]	Low	Low	Moderate	Low	Low	Low	Low
Facin et al., 2021 [22]	Moderate	Moderate	Low	Low	Moderate	Low	Moderate
Flores-García et al., 2022 [23]	Low	Low	Moderate	Low	Low	Low	Low
Franco-Marina et al., 2006 [24]	Low	Moderate	Moderate	Low	Moderate	Low	Moderate
Galván-Portillo et al., 2022 [25]	Low	Low	Moderate	Low	Low	Low	Low
Giacomazzi et al., 2023 [26]	Moderate	Moderate	Moderate	Low	Moderate	Low	Moderate
Hernández-Pérez et al., 2022 [27]	Low	Low	Moderate	Low	Low	Low	Low
Hernández-Ramírez et al., 2009 [28]	Low	Low	Low	Low	Low	Low	Low
Herrero et al., 1989 [29]	Moderate	Low	Moderate	Low	Low	Low	Moderate
Iscovich et al., 1987 [30]	Moderate	Moderate	Moderate	Low	Moderate	Low	Moderate
Jiménez-Mendoza et al., 2018 [31]	Low	Low	Moderate	Low	Low	Low	Low
Lajous et al., 2006 [32]	Moderate	Low	Low	Low	Low	Low	Low
López-Carrillo et al., 1994 [33]	Low	Moderate	Moderate	Low	Low	Low	Moderate
López-Carrillo et al., 1998 [34]	Low	Moderate	Moderate	Low	Low	Low	Moderate
López-Carrillo et al., 2003 [35]	Moderate	Low	Low	Low	Low	Low	Low
Luz et al., 2011 [36]	Moderate	Moderate	Moderate	Low	Moderate	Moderate	Moderate
Marchioni et al., 2007 [37]	Moderate	Low	Moderate	Low	Low	Low	Moderate
Navarro-Ibarra et al., 2015 [38]	Moderate	Moderate	Low	Low	Moderate	Low	Moderate
Okada et al., 2020 [39]	Moderate	Moderate	Moderate	Low	Moderate	Low	Moderate
Ortiz-Mendoza et al., 2013 [40]	Moderate	Moderate	Low	Low	High	Moderate	High
Pineda et al., 2024 [41]	Moderate	Moderate	Low	Low	Low	Low	Moderate

Queiroz et al., 2018 [42]	Moderate	Moderate	Moderate	Low	Moderate	Low	Moderate
Ramírez-Díaz et al., 2021 [43]	Moderate	Moderate	Moderate	Low	Moderate	Low	Moderate
Rodrigues et al., 2019 [44]	Moderate	Moderate	Moderate	Low	High	Moderate	High
Ronco et al., 2016 [45]	Moderate	Low	Moderate	Low	Low	Low	Moderate
Salazar-Martínez et al., 2000 [46]	Moderate	Low	Low	Low	Low	Low	Low
Salazar-Martínez et al., 2002 [47]	Moderate	Moderate	Low	Low	Moderate	Low	Moderate
Salazar-Martínez et al., 2005 [48]	Moderate	Low	Low	Low	Low	Low	Low
Sanabria-Rojas et al., 2017 [49]	Moderate	Moderate	High	Low	High	Moderate	High
Sánchez-Zamorano et al., 2019 [50]	Low	Low	Low	Low	Low	Low	Low
Sassano et al., 2024 [51]	Moderate	Moderate	Moderate	Low	Low	Low	Moderate
Shivappa et al., 2018 [52]	Low	Low	Moderate	Low	Low	Low	Low
Silva et al., 2023 [53]	Moderate	Moderate	Low	Low	Low	Low	Moderate
Strom et al., 1995 [54]	Moderate	Moderate	Moderate	Low	Moderate	Low	Moderate
Szymańska et al., 2011 [55]	Moderate	Low	Low	Low	Low	Low	Low
Toledo et al., 2010 [56]	Moderate	Low	Moderate	Low	Low	Low	Moderate
Toporcov et al., 2012 [57]	Moderate	Moderate	Moderate	Low	Low	Low	Moderate
Torres-Sánchez et al., 2000 [58]	Moderate	Moderate	Low	Low	Moderate	Low	Moderate
Torres-Sánchez et al., 2009 [59]	Moderate	Moderate	Low	Low	Low	Low	Moderate
Tumas et al., 2014 [60]	Low	Low	Moderate	Low	Moderate	Low	Low
Vázquez-Salas et al., 2016 [61]	Low	Low	Moderate	Low	Low	Low	Low

QUIPS domains: study participation, study attrition, prognostic-factor measurement, outcome measurement, study confounding, and statistical analysis & reporting. The overall rating is the highest (worst) level of concern across domains. López-Carrillo 2003 and Sassano 2024 are not included here (rated by analogy / consortium design).

Quality In Prognosis Studies tool applied to each included study. Domain and overall ratings: Low / Moderate / High.

Supplementary Table S2. Exploratory domain-level synthesis across cancer sites

Domain	k	Clusters (m)	OR (95% CI, CR2)	tau2 (total)	df
Healthy diet	9	9	0.42 (0.32-0.54)	0.028	6.4
Physical activity	3	3	not estimable (m<5; CI not interpretable)	0.000	
Adiposity	6	6	2.04 (0.80-5.21)	0.572	4.6
Unhealthy diet	16	15	1.88 (1.37-2.59)	0.239	13.2
Alcohol consumption	5	5	2.32 (1.00-5.36)	0.400	3.9

Cigarette smoking	7	5	3.06 (1.32-7.09)	0.414	3.9
**Healthy diet — exposure-definition sensitivity (direction of effect)**					
Composite dietary patterns only	4	4	4/4 protective; median OR 0.41; RE 0.35 (m<5: robust CI not reported)	—	—
Isolated nutrients / single foods only	5	5	5/5 protective; median OR 0.40; RE 0.40 (0.28–0.58)	—	—

CR2, bias-reduced cluster-robust variance estimator. For domains with fewer than five clusters (m < 5; physical activity, alcohol) the confidence interval is not reported (not estimable). These cross-site estimates are never a primary finding. k, number of estimates; m, number of clusters; τ^2, total between-cluster variance; df, Satterthwaite degrees of freedom.

NOT site-specific and NOT confirmatory — interpret with extreme caution. Combines heterogeneous exposures across different cancer sites; no causal interpretation. Exposure-definition sensitivity: within the healthy-diet domain, the protective direction was preserved whether the synthesis was restricted to composite dietary patterns (Denova-Gutiérrez 2014; Toledo 2010; Sanabria-Rojas 2017; Galván-Portillo 2022; 4/4 protective) or to isolated nutrients/single foods (Lajous 2006; Ronco 2016; Salazar-Martínez 2002; Torres-Sánchez 2000; Torres-Sánchez 2009; 5/5 protective). Because the composite-patterns subset has fewer than five clusters, its robust confidence interval is not reported.

Supplementary Table S3. Dose–response meta-analysis: DII/E-DII and prostate cancer

DII/E-DII tertile (rank)	Pooled OR (95% CI)
T1 (ref)	1.00 (1.00-1.00)
T2	1.18 (1.05-1.33)
T3	1.39 (1.10-1.76)
OR per tertile-step	1.18 (1.05-1.33)

Pooled odds ratios by DII/E-DII tertile relative to the lowest tertile (T1), and the linear trend per tertile-step. DII, dietary inflammatory index; E-DII, energy-adjusted DII; OR, odds ratio; CI, confidence interval.

Two-stage (Greenland–Longnecker) dose–response model; tertile rank as the dose metric (raw DII vs energy-adjusted E-DII scales differ).

Supplementary Table S4. E-values for unmeasured confounding

Study	Domain	Site	OR	95% CI	E-value (point)	E-value (CI)
Toledo 2010	Healthy diet	Oral	0.440	0.25-0.75	2.38	1.58
Ronco 2016	Healthy diet	Breast	0.380	0.25-0.58	2.63	1.95
TorresSanchez 2000	Healthy diet	Breast	0.270	0.16-0.47	3.26	2.28
Lajous 2006	Healthy diet	Breast	0.640	0.45-0.90	1.81	1.29
Rodrigues 2019	Healthy diet	Breast	0.410	0.20-0.84	2.50	1.41
Salazar Martinez 2002	Healthy diet	Ovarian	0.430	0.23-0.80	2.42	1.48

Denova Gutierrez 2014a	Healthy diet	Gastric	0.430	0.24-0.77	2.42	1.54
Galvan Portillo 2022	Healthy diet	Prostate	0.390	0.27-0.56	2.58	2.01
Sanabria 2017	Healthy diet	Colorectal	0.050	0.01-0.59	8.41	1.93
Coquet 2016	Unhealthy diet	Breast	1.300	1.00-1.70	1.54	1.00
Pineda 2022	Unhealthy diet	Breast	4.990	1.39-17.92	3.89	1.64
Denova Gutierrez 2014b	Unhealthy diet	Gastric	4.800	2.56-8.98	3.81	2.58
Queiroz 2018d	Unhealthy diet	Breast	2.350	1.08-5.12	2.44	1.24
Marchioni 2007	Unhealthy diet	Oral	1.490	0.99-2.27	1.74	1.00
Toporcov 2012	Unhealthy diet	Oral	3.040	1.51-6.15	2.88	1.76
Lopez Carrillo 1994	Unhealthy diet	Gastric	5.490	2.72-11.06	4.12	2.68
Lopez Carrillo 2003	Unhealthy diet	Gastric	1.710	0.76-3.88	1.94	1.00
Silva 2023	Unhealthy diet	Gastric	2.600	1.16-5.70	2.61	1.37
Shivappa 2018	Unhealthy diet	Prostate	1.500	1.24-1.80	1.75	1.47
Vazquez Salas 2016	Unhealthy diet	Prostate	1.180	0.85-1.63	1.39	1.00
Salazar Martinez 2002b	Unhealthy diet	Ovarian	0.600	0.33-1.06	1.90	1.00
Salazar Martinez 2005	Unhealthy diet	Endometrial	1.190	0.55-2.58	1.41	1.00
Amadou 2015	Unhealthy diet	Breast	1.300	1.00-1.70	1.54	1.00
Luz 2011	Unhealthy diet	Brain	1.300	0.61-2.78	1.54	1.00
Ramirez Diaz 2021	Unhealthy diet	Colorectal	2.790	1.32-5.89	2.73	1.56
Angeles Llerenas 2010	Physical activity	Breast	0.610	0.40-0.94	1.88	1.21
Facin 2021	Physical activity	Colorectal	0.830	0.31-2.28	1.43	1.00
Salazar Martinez 2000a	Physical activity	Endometrial	0.470	0.26-0.86	2.28	1.37
Franco Marina 2006	Cigarette smoking	Lung	4.000	2.90-5.50	3.41	2.80
Giacomazzi 2023a	Cigarette smoking	Breast	4.200	2.40-7.50	3.52	2.47
Giacomazzi 2023b	Cigarette smoking	Colorectal	3.100	1.50-6.30	2.92	1.75
Giacomazzi 2023c	Cigarette smoking	Prostate	10.500	4.20-26.30	5.93	3.52
Iscovich 1987	Cigarette smoking	Bladder	4.320	1.90-10.30	3.58	2.10
Herrero 1989	Cigarette smoking	Cervical	1.000	0.70-1.20	1.00	1.00
Castellsague 1999a	Cigarette smoking	Esophageal	3.240	2.40-4.37	3.00	2.47
Beasley 2010	Alcohol consumption	Breast	1.250	0.99-1.58	1.48	1.00
Okada 2020	Alcohol consumption	Colorectal	1.160	0.87-1.55	1.37	1.00
Szymanska 2011	Alcohol consumption	Oral	4.620	3.39-6.28	3.72	3.09
Lopez Carrillo 1998	Alcohol consumption	Gastric	2.930	1.27-6.75	2.82	1.51
Castellsague 1999b	Alcohol consumption	Esophageal	3.700	2.70-4.90	3.26	2.67
Amadou 2014	Adiposity	Breast	0.604	0.46-0.80	1.89	1.48
Queiroz 2018	Adiposity	Breast	2.700	1.28-5.70	2.67	1.52
Ortiz Mendoza 2013	Adiposity	Endometrial	8.100	2.46-26.60	5.14	2.51
Salazar Martinez 2000c	Adiposity	Endometrial	2.200	1.20-4.20	2.33	1.42
Hernandez Perez 2022	Adiposity	Prostate	1.940	1.37-2.75	2.13	1.62
Strom 1995	Adiposity	Gallbladder	2.600	0.50-18.60	2.61	1.00

E-value (point): for the point estimate. E-value (CI): for the confidence-interval limit closest to the null (E-value = 1.00 when the CI already crosses the null). Larger E-values indicate greater robustness to unmeasured confounding. OR, odds ratio; CI, confidence interval.

Minimum strength of association (on the risk-ratio scale) that an unmeasured confounder would need with both the exposure and the outcome to fully explain away the observed estimate.

Supplementary Table S5. Low-risk-of-bias sensitivity analysis (direction-of-effect synthesis)

Domain	Low-RoB k	Protective	Risk	Median OR	Direction vs full synthesis
Unhealthy diet	7	0	7	1·62	Unchanged (harmful)
Healthy diet	4	4	0	0·54	Unchanged (protective)
Cigarette smoking	1	0	1	2·30	Unchanged (harmful)
Alcohol consumption	2	0	2	3·09	Unchanged (harmful)
Physical activity	2	2	0	0·60	Unchanged (protective)
Adiposity	4	2	2	1·34	Risk-leaning but non-significant in full set; evenly split

Direction-of-effect synthesis restricted to studies rated low overall risk of bias (QUIPS). Counts are estimates; the final column compares the low-risk subset with the complete synthesis (Table 3). The direction of effect was preserved in every domain with a clear directional signal; adiposity was already non-directional in the full synthesis (binomial p = 0·22). Computed on the audited estimate set.

Data S1. Full electronic search strategy adapted to each database, related to STAR Methods.

Systematic review of lifestyle risk factors and cancer risk in Latin America (ISCIENCE-D-26-03890). Databases searched from inception to September 2025, with no language restriction. The search combines three concept blocks with the Boolean operator AND: (1) Latin American region/countries; (2) modifiable lifestyle exposures (smoking, alcohol, adiposity, physical activity, diet); and (3) cancer/neoplasms. Truncation (*) captures word variants. Reference lists of included studies were hand-searched. The exact syntax adapted to each database is reproduced below.

1. PubMed / MEDLINE (field tags: [MeSH], [tiab])

("Latin America"[MeSH] OR "South America"[MeSH] OR "Central America"[MeSH] OR Argentin*[tiab] OR Brazil*[tiab] OR Chile*[tiab] OR Colombia*[tiab] OR Mexic*[tiab] OR Peru*[tiab] OR Uruguay*[tiab] OR Venezuela*[tiab] OR Ecuador*[tiab] OR Bolivia*[tiab] OR Paraguay*[tiab] OR "Costa Rica"[tiab] OR Cuba*[tiab] OR Guatemala*[tiab] OR Honduras*[tiab] OR Nicaragua*[tiab] OR Panama*[tiab] OR "Puerto Rico"[tiab] OR "Dominican Republic"[tiab])

AND

("Life Style"[MeSH] OR "Smoking"[MeSH] OR "Tobacco"[MeSH] OR "Alcohol Drinking"[MeSH] OR "Obesity"[MeSH] OR "Body Mass Index"[MeSH] OR "Exercise"[MeSH] OR "Sedentary Behavior"[MeSH] OR "Diet"[MeSH] OR "Feeding Behavior"[MeSH] OR smoking[tiab] OR tobacco[tiab] OR alcohol[tiab] OR obesity[tiab] OR adiposity[tiab] OR "body mass index"[tiab] OR "physical activity"[tiab] OR "physical inactivity"[tiab] OR sedentar*[tiab] OR diet*[tiab] OR "dietary pattern*"[tiab] OR nutrition*[tiab])

AND

("Neoplasms"[MeSH] OR cancer*[tiab] OR neoplas*[tiab] OR tumor*[tiab] OR tumour*[tiab] OR carcinoma*[tiab] OR malignan*[tiab])

2. Scopus (TITLE-ABS-KEY)

TITLE-ABS-KEY("Latin America" OR "South America" OR "Central America" OR Argentin* OR Brazil* OR Chile* OR Colombia* OR Mexic* OR Peru* OR Uruguay* OR Venezuela* OR Ecuador* OR Bolivia* OR Paraguay* OR "Costa Rica" OR Cuba* OR Guatemala* OR Honduras* OR Nicaragua* OR Panama* OR "Puerto Rico" OR "Dominican Republic")

AND TITLE-ABS-KEY(lifestyle OR smoking OR tobacco OR alcohol OR obesity

OR adiposity OR "body mass index" OR "physical activity" OR "physical inactivity" OR sedentar* OR diet* OR "dietary pattern*" OR nutrition*)
AND TITLE-ABS-KEY(cancer* OR neoplas* OR tumor* OR tumour* OR carcinoma* OR malignan*)

3. Web of Science (Topic, TS=)

TS=("Latin America" OR "South America" OR "Central America" OR Argentin* OR Brazil* OR Chile* OR Colombia* OR Mexic* OR Peru* OR Uruguay* OR Venezuela* OR Ecuador* OR Bolivia* OR Paraguay* OR "Costa Rica" OR Cuba* OR Guatemala* OR Honduras* OR Nicaragua* OR Panama* OR "Puerto Rico" OR "Dominican Republic")
AND TS=(lifestyle OR smoking OR tobacco OR alcohol OR obesity OR adiposity OR "body mass index" OR "physical activity" OR "physical inactivity" OR sedentar* OR diet* OR "dietary pattern*" OR nutrition*)
AND TS=(cancer* OR neoplas* OR tumor* OR tumour* OR carcinoma* OR malignan*)

4. LILACS (via iAHx Regional Portal; bilingual EN/ES/PT, tw: = words)

(tw:("Latin America" OR Latinoam* OR Argentin* OR Brasil* OR Brazil* OR Chile* OR Colombia* OR Mexic* OR México* OR Peru* OR Perú* OR Uruguay* OR Venezuela* OR Ecuador* OR Bolivia* OR Paraguay*))
AND (tw:("estilo de vida" OR lifestyle OR tabaco OR tabagismo OR smoking OR fumar OR alcohol OR álcool OR obesidad OR obesidade OR obesity OR "índice de masa corporal" OR "actividad física" OR "atividade física" OR "physical activity" OR sedentar* OR dieta OR diet* OR alimentação OR nutrición OR nutrição))
AND (tw:(cáncer OR câncer OR cancer OR neoplasia* OR neoplasm* OR tumor* OR carcinoma* OR maligno*))

Notes. The PRISMA 2020 flow diagram and checklist accompany the manuscript. Records were de-duplicated in a reference manager; titles/abstracts and then full texts were screened in duplicate against the pre-specified inclusion criteria. This file is cited in the STAR Methods (Search strategy and selection criteria).
